# Late Endosomes Act as mRNA Translation Platforms and Sustain Mitochondria in Axons

**DOI:** 10.1016/j.cell.2018.11.030

**Published:** 2019-01-10

**Authors:** Jean-Michel Cioni, Julie Qiaojin Lin, Anne V. Holtermann, Max Koppers, Maximilian A.H. Jakobs, Afnan Azizi, Benita Turner-Bridger, Toshiaki Shigeoka, Kristian Franze, William A. Harris, Christine E. Holt

**Affiliations:** 1 Department of Physiology, Development and Neuroscience, University of Cambridge, Cambridge CB2 3DY, UK

**Keywords:** RNA trafficking, mRNA translation, endosome, lysosome, local protein synthesis, mitochondria, axon maintenance, retinal ganglion cell, Charcot-Marie-Tooth type 2B

## Abstract

Local translation regulates the axonal proteome, playing an important role in neuronal wiring and axon maintenance. How axonal mRNAs are localized to specific subcellular sites for translation, however, is not understood. Here we report that RNA granules associate with endosomes along the axons of retinal ganglion cells. RNA-bearing Rab7a late endosomes also associate with ribosomes, and real-time translation imaging reveals that they are sites of local protein synthesis. We show that RNA-bearing late endosomes often pause on mitochondria and that mRNAs encoding proteins for mitochondrial function are translated on Rab7a endosomes. Disruption of Rab7a function with Rab7a mutants, including those associated with Charcot-Marie-Tooth type 2B neuropathy, markedly decreases axonal protein synthesis, impairs mitochondrial function, and compromises axonal viability. Our findings thus reveal that late endosomes interact with RNA granules, translation machinery, and mitochondria and suggest that they serve as sites for regulating the supply of nascent pro-survival proteins in axons.

## Highlights

• Ribonucleoprotein particles are associated with endosomes in axons
• Rab7a endosomes provide sites for axonal local translation
• Rab7a endosomes support axonal synthesis of survival factors
• CMT2B-Rab7a mutations affect axonal translation and mitochondrial integrity

## Graphical Abstract

**Figure:**
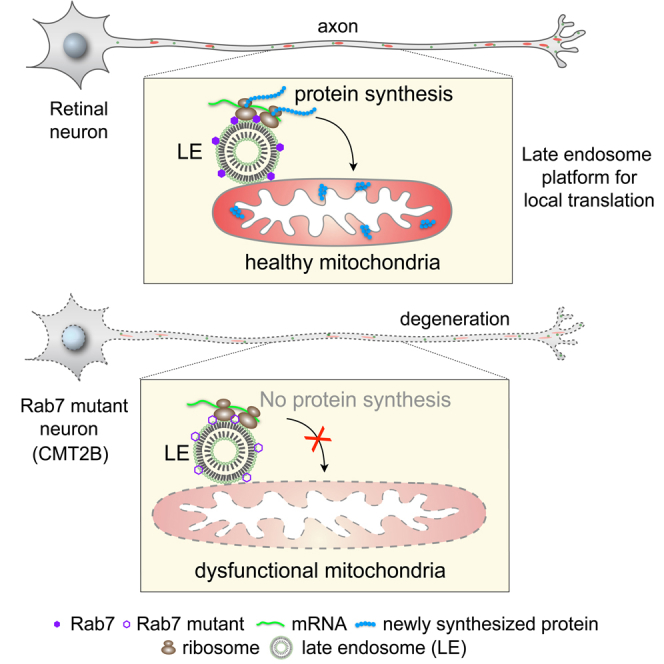

## Teaser

Late endosomes are sites of local translation of mRNAs important for mitochondrial maintenance, and perturbing this process can contribute to neuropathology, as seen in Charcot Marie-Tooth disease.

## Introduction

Local translation of mRNAs is a highly conserved mechanism that allows spatial and temporal control of the proteome at a subcellular level (Martin and Ephrussi, 2009). This regulation is particularly important in neurons, where tight control of protein localization allows highly compartmentalized functions far from the cell soma (Holt and Schuman, 2013, Terenzio et al., 2017). mRNAs made in the nucleus associate directly with RNA-binding proteins (RBPs) (Andreassi and Riccio, 2009, Xing and Bassell, 2013) and are transported as ribonucleoprotein particles (RNPs) to distal subcellular locations for local translation (Eliscovich and Singer, 2017, Mitchell and Parker, 2014, Xing and Bassell, 2013). However, the mechanisms regulating precise RNP localization and nascent protein synthesis at defined neuronal sites remain largely unknown.

The endosomal pathway has been implicated in mRNA localization and translation in the fungus *Ustilago maydis*, where the polarized growth of long hyphae requires endosomal-dependent transport of the RBP, Rrm4, and translation of its cargo *septin* mRNA (Baumann et al., 2014). In neurons, membrane trafficking relies on endosomes, which carry a range of proteins and lipids for targeted delivery (Cosker and Segal, 2014, Lasiecka and Winckler, 2011). The endosomal pathway internalizes cargos from the cell surface, regulates their storage and their recycling, or sends them to lysosomes for degradation (Huotari and Helenius, 2011). In addition to their role in trafficking, endosomes operate as platforms where diverse intracellular signaling cascades can be activated or sustained (Villaseñor et al., 2016). Two of the main players of this endosomal system are the early and late endosomes that can be distinguished by their associated Rab guanosine triphosphatases (GTPases) (Stenmark, 2009); Rab5 coordinates clathrin-dependent endocytosis and biogenesis of early endosomes and their fusion, whereas Rab7 regulates the transport and maturation of acidic late endosomes as well as their fusion with lysosomes.

Here we show that RNPs associate with motile Rab7a endosomes along retinal ganglion cell (RGC) axons. RNP-bearing Rab7a endosomes frequently dock at mitochondria, where they serve as hotspots for *de novo* protein synthesis. Disruption of Rab7a function by expression of Charcot-Marie-Tooth disease type 2B (CMT2B)-linked Rab7a mutants leads to impaired local protein synthesis, mitochondrial dysfunction, and loss of axon integrity.

## Results

### RNA Granules Are Associated with Endosomes in Axons

**Figure 1:**
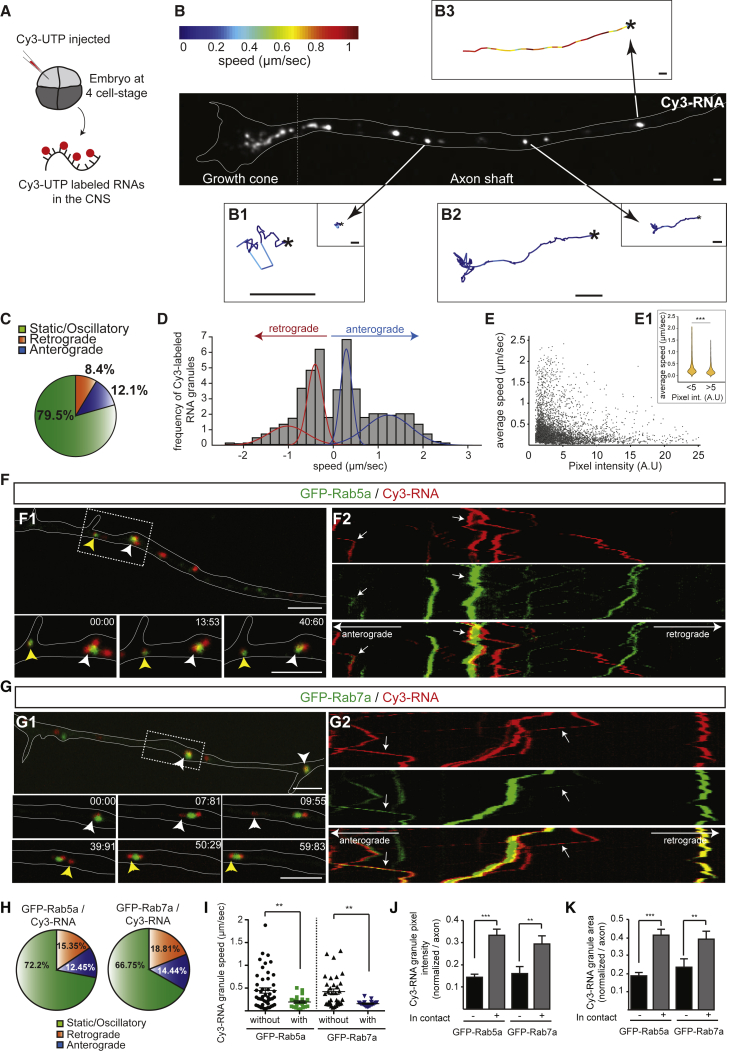
RNA Granules Are Associated with Endosomes in Axons (A) Schematic of labeling endogenous RNAs in the *Xenopus* CNS. (B) RGC axon shaft and growth cone containing Cy3-RNA granules undergoing oscillatory motions (B1), slow movement (B2), and fast movement (B3). Asterisks show the origin of the tracks. The frame-to-frame speeds are indicated by color code. The tracks are presented first at the same magnification and then at higher magnification for (B1) and (B2). (C) Proportions of axonal Cy3-RNA granules displaying the indicated motion types. (D) Speed distribution from average velocities of moving Cy3-RNA granules showing fast-moving and slow-moving populations in both anterograde (blue) and retrograde (red) directions (Gaussian mixture model). n = 1,022 moving RNA granules in 38 axons. (E) Scatterplot showing individual Cy3-RNA granule speed as a function of fluorescent pixel intensity. (E1) Violin plot showing the speed distribution of Cy3-RNA granules with pixel intensity either more or less than 5 a.u. (C–E) n = 4,995 RNA granules in 38 axons. (F) RGC axon segment showing the association between Cy3-RNA granules (red) and GFP-Rab5a (green) signals (white and yellow arrowheads indicate two different RNA granules) (F1). Also shown are kymographs (1 min) of the axon segment presented in (F1) (F2). (G) RGC axon segment showing the close association between Cy3-RNA granules (red) and GFP-Rab7a (green) signals (white and yellow arrowheads indicate two different RNA granules) (G1). Also shown are kymographs (1 min) of the axon segment presented in (G1) (G2). (H) Proportions of Cy3-RNA granules associated with GFP-Rab5a or GFP-Rab7a displaying the indicated motions (n = 52 [GFP-Rab5a] and n = 56 [GFP-Rab7a] RNA granules). (I) Speed of Cy3-RNA granules moving with or without GFP-Rab5a or GFP-Rab7a endosomes in axon shafts (n = 71 [GFP-Rab5a] and n = 52 [GFP-Rab7a] RNA granules). (J and K) Average pixel intensity (normalized to the brightest pixel within each axon) (J) and area (normalized to the area of the largest granule) (K) of Cy3-RNA granules away from or in contact with GFP-Rab5a or GFP-Rab7a endosomes (n = 192 [GFP-Rab5a] and n = 82 [GFP-Rab7a] RNA granules). Mean ± SEM; ^∗∗^p < 0.01, ^∗∗∗^p < 0.001; Wilcoxon rank-sum test in (E), Mann-Whitney test in (I)–(K). Scale bars, 1 μm in (B) and 5 μm in (F) and (G). Time stamps are in the format of seconds:milliseconds. See also Figures S1 and S2 and Video S1.

RBPs and ribosomes associate with motile endosomes in fungal hyphae (Baumann et al., 2014, Higuchi et al., 2014), raising the possibility that endosomes are involved in RNA granule trafficking in other cell types with elongated profiles, such as vertebrate neurons. To visualize the movement of RNPs in the axons of *Xenopus* RGCs, we labeled endogenous RNAs by blastomere injection of the fluorescently labeled uridine-5′-triphosphate (Cy3-UTP). Cy3-UTP is incorporated into RNAs during synthesis, including rRNAs and mRNAs (Wong et al., 2017), allowing subsequent visualization of fluorescent Cy3-RNA granules in RGC axons of cultured embryonic eyes (Figure 1A). Single-particle tracking analysis revealed the presence of static or oscillatory (Figure 1B1), slow-moving (Figure 1B2), and fast-moving (Figure 1B3) states of RNA granule movements, with occasional switches from one state to another. Most Cy3-RNA granules displayed static or oscillatory motions over 2 min (Figure 1C), similar to single mRNAs in dendrites (Yoon et al., 2016). A slight bias toward anterograde compared with retrograde transport (Figure 1C) is consistent with the directional movements of Neurofilament-Light (*Nefl*) mRNA in axons (Alami et al., 2014). The speed distribution of transported RNA granules could be resolved into two classes of anterograde or retrograde transport: slow and fast (Figure 1D), as observed for endogenous *β-actin* mRNA in axons (Turner-Bridger et al., 2018). The average speed of individual Cy3-RNA granules was negatively correlated with their signal intensities, suggesting that slow-moving or static granules can carry a larger RNA cargo load (Figure 1E).

**Figure S1:**
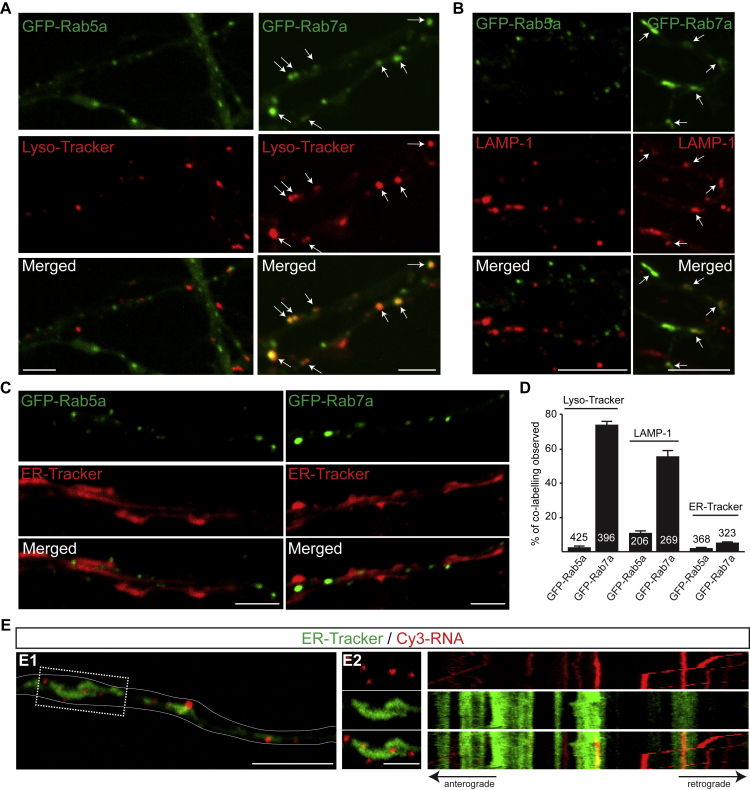
Spatial Relationship between Early Endosomes, Late Endosomes, the ER, and Cy3-RNA Granules in Cultured RGC Axons, Related to Figure 1 (A) GFP-Rab5a endosomes do not colocalize with Lyso-Tracker-labeled vesicles in distal axons (left). GFP-Rab7a endosomes colocalize with Lyso-Tracker-labeled vesicles (right, white arrows). (B) GFP-Rab5a endosomes do not colocalize with LAMP-1-associated vesicles in distal axons (left). GFP-Rab7a endosomes colocalize with LAMP-1-associated vesicles (right, white arrows). (C) GFP-Rab5a and GFP-Rab7a endosomes are in close contact with ER-Tracker-labeled axonal ER, but they are structurally distinct. (D) Percentage of GFP-Rab5a or GFP-Rab7a endosomes co-labeled with Lyso-Tracker, LAMP-1 or ER-Tracker. (E) Cy3-RNA granules are closely associated with ER-Tracker-labeled axonal ER (left). Kymograph of the axon segment indicated by the dotted outline (middle) shows little co-movement of the two signals (right). n, number of axon segments. Mean ± s.e.m. Scale bars: 5 μm in A, B, C, D, E1; 2 μm in E2.

To find out whether RNA granules in axons are associated with endosomes, we used GFP-Rab5a or GFP-Rab7a, which label early and late endosomes, respectively (Falk et al., 2014; Figures S1A–S1D). Both exhibited typically rounded profiles, with occasional variations in size and shape indicative of different endosomal maturation and/or fusion states. By expressing GFP-Rab5a and GFP-Rab7a along with Cy3-labeled RNAs, we found that static, oscillatory, and moving Cy3-RNA granules were often associated with both early and late endosome reporters in axons (Figures 1F and 1G; Video S1). RNA granules appeared to associate and dissociate from endosomes (Figure 1G), a characteristic similar to that observed for RNPs in fungal hyphae (Higuchi et al., 2014). Cy3-RNA granules were preferentially associated with GFP-Rab7a (40% ± 3%, n = 298 RNA granules in 23 axons) compared with GFP-Rab5a (24% ± 1%, n = 347 RNA granules in 24 axons). Despite the abundance of endoplasmic reticula (ERs) throughout the axon, the levels of co-labeling between Rab5a or 7a endosomes, and ERs was comparatively low (Figures S1C and S1D), and dynamic co-imaging showed that Cy3-RNA granules moved independently of the ER (Figure S1E). Static or oscillatory movements were most prevalent among RNA granules associated with Rab5a or Rab7a endosomes (Figure 1H). Furthermore, RNA granules associated with GFP-Rab5a or GFP-Rab7a endosomes moved significantly slower than isolated RNA granules in the same axons (Figure 1I). The average pixel intensity and size of endosome-associated Cy3-RNA granules were higher and larger than those of isolated Cy3-RNA granules, suggesting the presence of more RNA (Figures 1J and 1K).

Video S1. Association of Cy3-RNA Granules (Red) with GFP-Rab5a or GFP-Rab7a Endosomes (Green), Related to Figure 1

**Figure S2:**
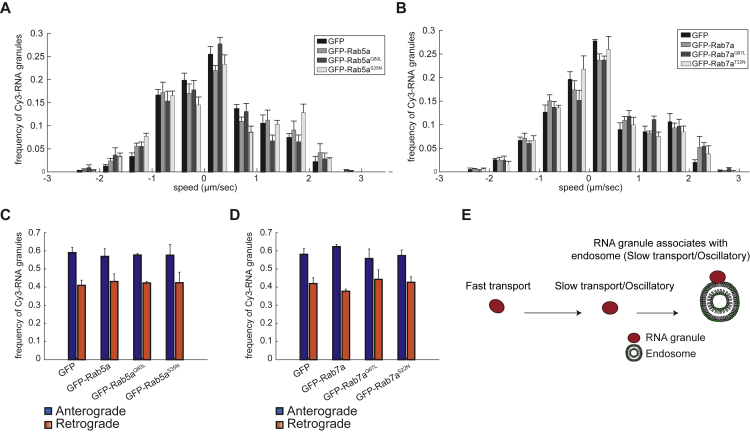
RNA Granule Motions in Axons Expressing Wild-Type or Mutant GFP-Rab5a or GFP-Rab7a, Related to Figure 1 (A) Average speed of Cy3-RNA granules in axons expressing GFP, GFP-tagged wild-type Rab5a (Rab5a) or Rab5a mutants (Rab5a^Q80L^, Rab5a^S35N^). n=547 (GFP), 1064 (GFP-Rab5a), 695 (GFP-Rab5a^Q80L^), 524 (GFP-Rab5a^S35N^) granules. (B) Average speed of Cy3-RNA granules in axons expressing GFP, GFP-tagged wild-type Rab7a (Rab7a) or Rab7a mutants (Rab7a^Q67L^, Rab7a^T22N^). n=475 (GFP), 788 (GFP-Rab7a), 657 (GFP-Rab7a^Q67L^), and 811 (GFP-Rab7a^T22N^) granules. (A and B) For each of the three biological replicates we calculated a frequency distribution of speeds for the following bins: -2.5 to -2, -2 to -1.5, -1.5 to -1, -1 to -0.5,-0.5 to 0, 0 to 0.5, 0.5 to 1, 1 to 1.5, 1.5 to 2, 2 to 2.5, and 2.5 to 3. For each bin the bar plot represents the mean of the 3 replicates. (C) Proportions of Cy3-RNA granules transported anterogradely or retrogradely in axons expressing GFP as control, GFP-tagged wild-type Rab5a or Rab5a mutants Rab5a^Q80L^, Rab5a^S35N^. Data from all repeats are pooled. n=21 (GFP), 18 (GFP-Rab5a, 16 (GFP-Rab5a^Q80L^), 17 (GFP-Rab5a^S35N^) axons. (D) Proportions of Cy3-RNA granules transported anterogradely or retrogradely in axons expressing GFP as control (CT), GFP-tagged wild-type Rab7a (WT) or Rab7a mutants Rab7a^Q67L^, Rab7a^T22N^. Data from all repeats are pooled. n=17 (GFP), 18 (GFP-Rab7a), 20 (GFP-Rab7a^Q67L^), 20 (GFP-Rab7a^T22N^) axons. Mean±s.e.m.

We next asked whether Rab activity plays a role in global RNA granule trafficking in RGC axons. Expression of constitutively active (CA) or dominant-negative (DN) forms of Rab5a, but not Rab7a, induced a visible reduction in axonal growth, as reported previously (Falk et al., 2014). However, expression of mutant forms of either Rab5a or Rab7a did not affect the RNA granule speed distribution profile or directionality compared with controls (Figures S2A–S2D).

Collectively, these results suggest that, although RNA granules are mostly transported and distributed in axons in an endosome-independent manner, they frequently associate with both early and late endosomes along the axon (Figure S2E).

### Ribosomes, RBPs, and mRNAs Localize to Endosomes in Axons

**Figure 2:**
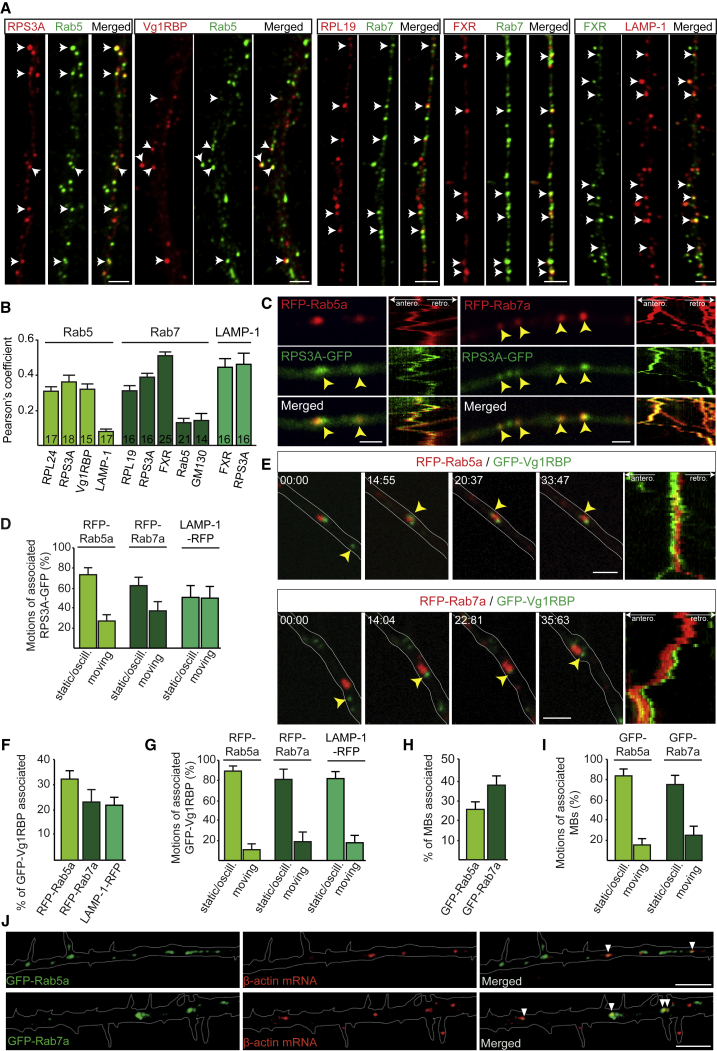
Ribosomes, RNA-Binding Proteins, and mRNAs Localize to Endosomes in Axons (A) Colocalization between endosomal markers and RNA-binding proteins (RBPs) or ribosomal proteins. (B) Pearson’s coefficient between endosomal markers and RBPs or ribosomal proteins. n, number of axon segments. (C) Time-lapse images and kymographs (1 min) illustrating RPS3A-GFP association with RFP-Rab5a or Rab7a endosomes (yellow arrowheads) in axons. (D) Motion types of RFP-tagged Rab5a (n = 27 axons), Rab7a (n = 29 axons), or LAMP-1 (n = 22 axons) endosomes associated with the RPS3A-GFP signal. (E) Time-lapse images and kymographs illustrating GFP-Vg1RBP association with RFP-Rab5a or Rab7a endosomes (yellow arrowheads) in axons. (F and G) Frequency (F) and motion types (G) of GFP-Vg1RBP associated with RFP-tagged Rab5a (n = 15 axons), Rab7a (n = 13 axons), or LAMP-1 (n = 12 axons) endosomes. (H and I) Frequency (H) and motion types (I) of *β-actin* molecular beacon (MB) signals associated with GFP-Rab5a (n = 22 axons) or GFP-Rab7a (n = 20 axons) endosomes. (J) Representative images showing *β-actin* MBs associated with GFP-Rab5a or GFP-Rab7a endosomes (white arrowheads) in axons. Mean ± SEM. Scale bars: 2.5 μm in (A), 2 μm in (C) and (E), and 5 μm in (J). Time stamps are in the format of seconds:milliseconds. See also Figure S3 and Videos S2 and S3.

Previous mass spectrometry analysis of endosomal composition in neurons revealed the presence of RBPs and translation machinery (Debaisieux et al., 2016). We therefore performed immunocytochemistry against known RNP components in RGC axons (Cioni et al., 2018, Leung et al., 2006). Rab5, Rab7, and the late endosome marker LAMP-1 partially colocalized with RBPs such as Vg1RBP (zipcode-binding protein-1) or the *Xenopus* RBP Fragile X-related (FXR) as well as ribosomal proteins in axon shafts (Figures 2A and 2B).

**Figure S3:**
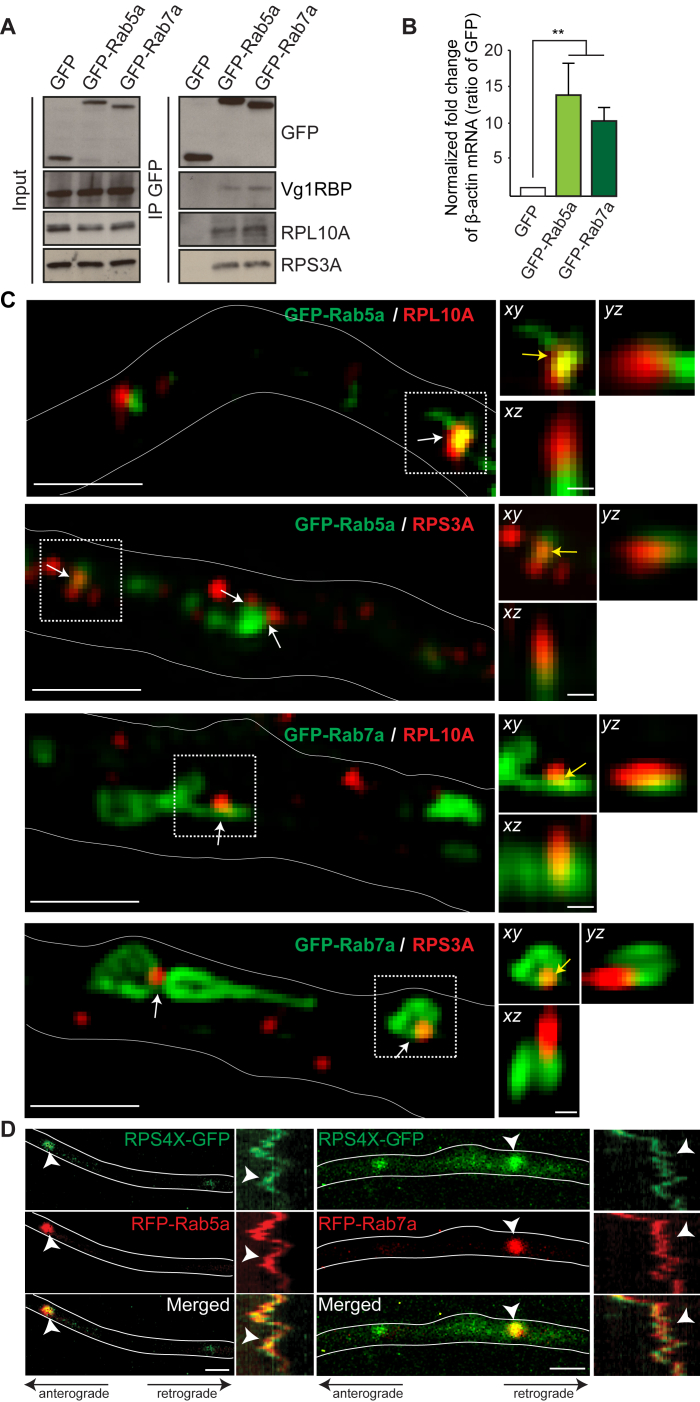
Ribosomes Associate with GFP-Rab5a and GFP-Rab7a Endosomes in Axons, Related to Figure 2 (A) Representative Western Blot of Vg1RBP and ribosomal proteins RPL10A and RPS3A co-immunoprecipitated with GFP-Rab5a and GFP-Rab7a in brain lysates. (B) qRT-PCR of cDNA synthesized from RNAs co-precipitated with GFP, GFP-Rab5a and GFP-Rab7a in brain lysates revealing a significant enrichment of *β-actin* mRNA (n=3 biological replicates). (C) OMX Super-resolution microscopy revealing the presence of RPL10A and RPS3A puncta on GFP-Rab5a or GFP-Rab7a endosomes in axons (left panels, white arrows). Orthogonal views of the indicated areas confirming the contact (yellow arrows) between the ribosomal proteins and endosomes (right panels). (D) Time-lapse images and kymographs illustrating RPS4X-GFP association with RFP-Rab5a or Rab7a endosomes (white arrowheads) in axons. Mean±s.e.m.; ^∗∗^P<0.01, Mann-Whitney test. Scale bars: 1μm in left panels in C and D; 200nm in right panels in C.

To study these associations in more detail, we analyzed ribosomal proteins, Vg1RBP, and its *β-actin* mRNA cargo (Leung et al., 2006, Welshhans and Bassell, 2011) using live imaging. Ribosomal proteins, Vg1RBP, and *β-actin* mRNA each associate with GFP-tagged Rab5a and Rab7a proteins, as seen using immunoprecipitation (IP) assays (Figures S3A and S3B), and super-resolution microscopy revealed that ribosomal proteins closely associated with GFP-Rab5a and GFP-Rab7a endosomes (Figure S3C). The ribosomal protein RPS3A-GFP exhibited a mostly diffuse pattern of fluorescence interspersed with foci of higher intensity along the axon shaft. Live co-imaging of red fluorescent protein (RFP)-Rab5a, RFP-Rab7a, LAMP-1-RFP, and RPS3A-GFP revealed an association between the ribosomal and endosomal markers (Figure 2C; Video S2) that exhibited coordinated motions, mostly oscillatory or static, that persisted throughout the 1-min imaging period (Figure 2D). Similar results were obtained by co-expressing another ribosomal protein, RPS4X-GFP, with RFP-Rab5a or RFP-Rab7a (Figure S3D). Although the majority of the GFP-Vg1RBP signal was not co-transported with endosomes, we found that a fraction of GFP-Vg1RBP also joined slow-moving endosomes over the 1-min recording periods (Figures 2E and 2F; Video S3) and displayed static or oscillatory motions (Figure 2G). To track endogenous *β-actin* mRNA directly, we used Cy3-molecular beacons (MBs) (Turner-Bridger et al., 2018). MBs are hairpin-shaped oligonucleotide probes with a fluorophore and a quencher that separate upon hybridization to target sequences (Alami et al., 2014), allowing visualization of endogenous transcripts. MB tracking revealed that approximately 25% and 35% of *β-actin* mRNA granules were, indeed, associated with GFP-Rab5a and GFP-Rab7a (Figures 2H and 2J), of which most were static or oscillatory (Figure 2I). Together, these results indicate that ribosomes, RBPs, and mRNAs frequently assemble together with endosomes in axons.

Video S2. Association of RPS3A-GFP (Green) with RFP-Rab5a or RFP-Rab7a Endosomes (Red), Related to Figure 2

Video S3. Association of GFP-Vg1RBP (Green) with RFP-Rab5a or RFP-Rab7a Endosomes (Red), Related to Figure 2

### Late Endosomes Are Sites of Intra-axonal Protein Synthesis

**Figure 3:**
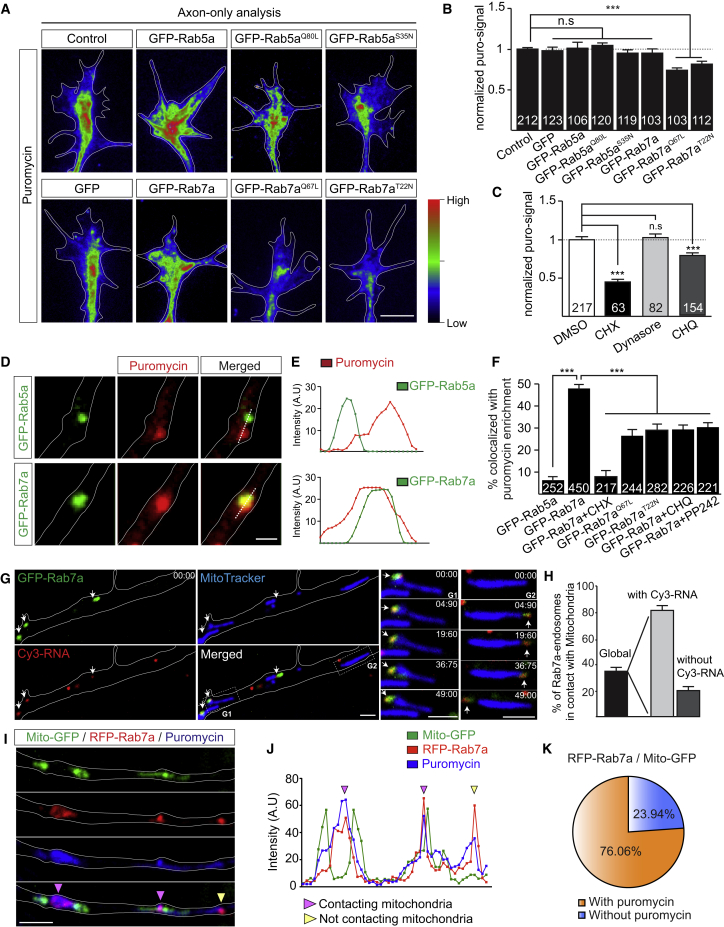
Perturbed Rab7a Activity and Late Endosome Maturation Reduced Intra-axonal Protein Synthesis (A) Heatmaps indicating relative puromycin fluorescence intensity (puro-signal) in somaless RGC growth cones. (B) Quantitative immunofluorescence (QIF) analysis of puromycin incorporation in somaless growth cones expressing the indicated constructs. (C) QIF analysis of puromycin incorporation in somaless growth cones upon acute pharmacological treatments. (D) Nascent proteins labeled by puromycin colocalizing with GFP-Rab7a endosomes but not GFP-Rab5a endosomes. (E) Plotted fluorescent signals across the dotted line in (D). (F) Percentage of GFP-tagged Rab5a-, Rab7a- and Rab7a mutant endosomes colocalizing with puromycin enrichment in axons. (G) Close association of GFP-Rab7a endosomes (green), Cy3-RNA granules (red), and mitochondria (blue) in RGC axons. White arrows indicate Cy3-RNA granules associated with GFP-Rab7a endosomes. (G1 and G2) Examples of time-lapse sequences showing Cy3-RNA granules associated with GFP-Rab7a endosomes next to mitochondria. (H) Percentage of GFP-Rab7a endosomes in contact with mitochondria, 80% of which are associated with Cy3-RNA granules (n = 335 GFP-Rab7a endosomes in 32 axons). (I) RGC axon segments expressing Mito-GFP (green) and RFP-Rab7a (red), in which newly synthesized proteins are visualized using puromycin labeling (blue). The puromycin signal is enriched on RFP-Rab7a endosomes in proximity to mitochondria (pink arrowheads) and distant from mitochondria (yellow arrowhead). (J) Plotted fluorescent signals along the axon segment presented in (I). (K) Percentage of RFP-Rab7a endosomes in contact with mitochondria with or without puromycin enrichment in axons (n = 52 axons). Mean ± SEM; N, number of growth cones in (B) and (C) or number of endosomes in (F). n.s., not significant. ^∗^p < 0.05, ^∗∗∗^p < 0.001, Mann-Whitney test. Scale bars, 5 μm in (A) and 2 μm in (D), (G), and (I). Time stamps are in the format of seconds:milliseconds. See also Figures S4 and S5.

Static RNA granules labeled with fluorescent UTPs are hotspots of newly synthesized proteins in axon terminals *in vivo* (Wong et al., 2017). Our findings above raise the possibility that these hotspots are on endosomes. To test this idea, we first investigated whether *de novo* protein synthesis in RGC axons is affected by altered Rab function. Axon-only cultures (after soma removal) were pulse-labeled with a low concentration of puromycin (2 μM), a structural analog of aminoacyl-tRNAs that incorporates into the C termini of nascent polypeptide chains. Puromycylated peptides can be recognized by anti-puromycin antibodies, allowing quantification of local protein synthesis (Schmidt et al., 2009). We found a significant reduction in puromycin signal in growth cones expressing CA mutant GFP-Rab7a^Q67L^ (0.74 ± 0.03) or DN mutant GFP-Rab7a^T22N^ (0.81 ± 0.04) but not wild-type GFP-Rab7a, wild-type GFP-Rab5a, CA mutant GFP-Rab5a^Q80L^, or DN mutant GFP-Rab5a^S35N^ (Figures 3A and 3B). Next, we acutely perturbed endosomal sorting pharmacologically (Figure 3C). Inhibition of endocytosis with dynasore, a small GTPase inhibitor targeting dynamin, for 20 min did not affect the puromycin signal, whereas the late endosomal acidification inhibitor chloroquine (CHQ) significantly attenuated it. The reduction in puromycin signal following CHQ treatment or with Rab7a mutant expression in axons was not as severe as that following treatment with the protein synthesis inhibitor cycloheximide (CHX) (Figure 3C), indicating that axonal protein synthesis is not exclusively endosome-dependent. We also saw a clear enrichment of the puromycin signal associated with GFP-Rab7a but not GFP-Rab5a endosomes (Figures 3D–3F), which was abolished by CHX treatment (Figure 3F). This enriched puromycin signal was also reduced by GFP-Rab7a^Q67L^ or GFP-Rab7a^T22N^ (Figure 3F) or application of CHQ (Figure 3F), confirming that both Rab7a activity and late endosome maturation are essential for late endosome-sited translation.

**Figure S4:**
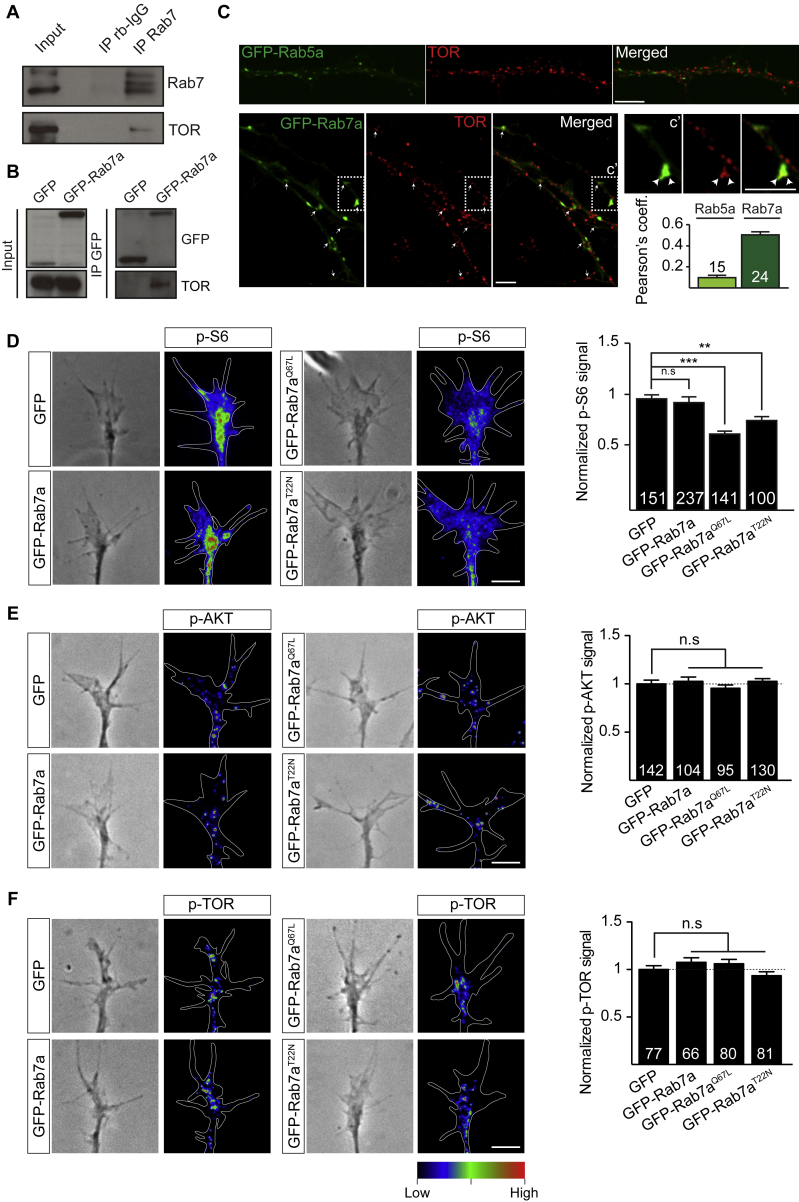
Perturbing Rab7a Function Disrupts Signaling Downstream of TOR Complex 1 in RGC Axons, Related to Figure 3 (A) Co-immunoprecipitation between endogenous Rab7 and TOR in brain lysates. (B) Co-immunoprecipitation between GFP-Rab7a and TOR in brain lysates. (C) Representative immunocytochemistry images showing TOR signals in RGC axons expressing GFP-Rab5a or GFP-Rab7a. Pearson’s coefficient between endosomal markers and TOR. (D) Representative phase contrast images and heat maps indicating relative phospho-S6 levels in growth cones expressing Rab7a mutants. GFP-Rab7a^Q67L^ or GFP-Rab7a^T22N^ expression decreases the amount of phospho-S6 ribosomal proteins in growth cones compared to GFP or GFP-Rab7a-expressing growth cones. (E) Representative phase contrast images and heat maps indicating relative phospho-AKT levels in growth cones expressing Rab7a mutants. GFP-Rab7a, GFP-Rab7a^Q67L^ or GFP-Rab7a^T22N^ expression does not affect the amount of phospho-AKT in growth cones compared to the GFP control. (F) Representative phase contrast images and heat maps indicating relative phospho-TOR levels in growth cones expressing Rab7a mutants. GFP-Rab7a, GFP-Rab7a^Q67L^ or GFP-Rab7a^T22N^ expression does not affect the amount of phospho-TOR in growth cones compared to the GFP control. n=number of axon segments in C, or n=number of growth cones in D, E, F. Mean±s.e.m.; n.s., not significant, ^∗∗^P<0.01, ^∗∗∗^P<0.001, Mann-Whitney test. Scale bars: 5μm.

Axonal protein synthesis is regulated by target of rapamycin complex 1 (TORC1) cap-dependent translation initiation (Campbell and Holt, 2001). Application of the TOR inhibitor PP242 significantly reduced late endosome-sited protein synthesis (Figure 3F). We found that TOR co-precipitated with endogenous Rab7 (Figure S4A) and GFP-Rab7a in brain lysates (Figure S4B) and colocalized with GFP-Rab7a in RGC axons (Figure S4C). We then measured phosphorylated ribosomal protein S6 (p-S6), a marker of TOR-mediated translation activity, in Rab7 mutant axons. Expression of GFP-Rab7a^Q67L^ or GFP-Rab7a^T22N^ mutants led to a decrease in p-S6 levels in axons compared with GFP-Rab7a and GFP (Figure S4D) without affecting the upstream TOR activation pathways (Figures S4E and S4F).

### Mitochondria Reside at Endosomal Translation Hotspots

**Figure S5:**
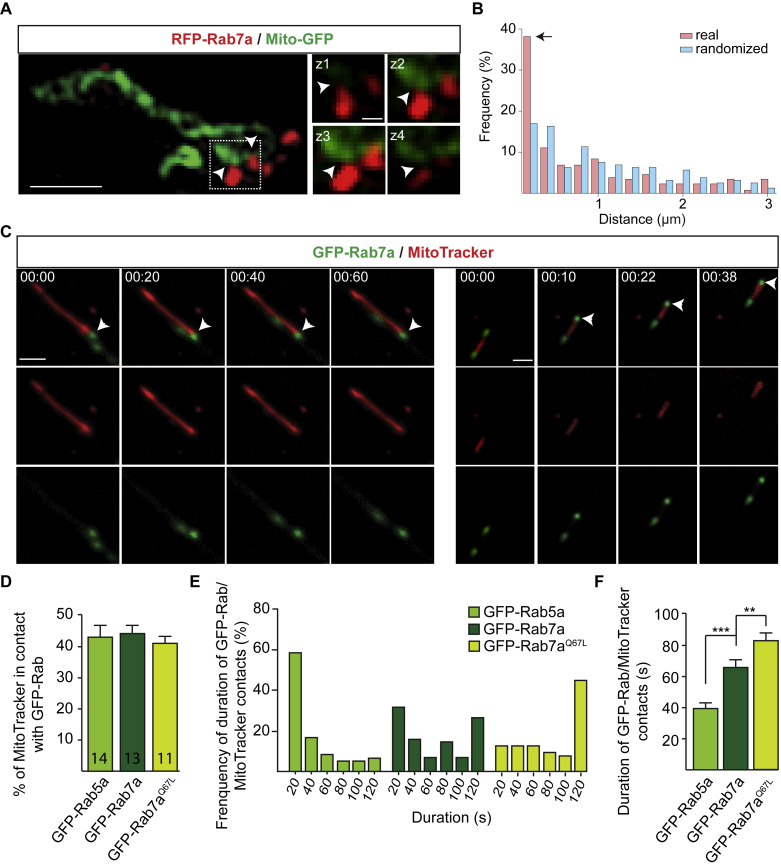
Endosomes and Mitochondria Form Contacts in RGC Axons, Related to Figure 3 (A) Representative super-resolution microscopy images showing the close proximity between RFP-Rab7a endosomes and mitochondria (Mito-GFP) in RGC axons. Images in z-stacks showing the close proximity between the two signals in the Z-plane. Arrowheads indicate the close proximity between the two signals. (B) Distribution of distances between real or randomly distributed RFP-Rab7a endosomes and mitochondria (Mito-GFP). The distance of each endosome with its nearest mitochondria is shown as a fraction of the total number of endosomes. The arrow indicates that the largest number of endosomes lie between 0-200 nm of the closest mitochondria (n=188 endosomes in 21 axons). (C) Examples of time-lapse sequences showing the contact between GFP-Rab7a endosomes and mitochondria (MitoTracker) in RGC axons. (D) Percentage of GFP-Rab5a or GFP-Rab7a endosomes contacting mitochondria for more than 10 seconds over 2-minute recording (n=number of axon segments). (E and F) Duration of endosome-mitochondria contacts over 2-minute recording (n=77 (GFP-Rab7a), 60 (GFP-Rab5a), 63 (GFP-Rab7a^Q67L^) contacts). Mean±s.e.m.; n.s., not significant, ^∗∗∗^P<0.001, Mann-Whitney test. Scale bars: 1μm (left panel) or 200nm (z-stacks) in A; 2.5μm in C. Time stamps are in the format of min:sec.

Mitochondrial respiration helps to sustain protein synthesis, and, in axons, focal translation hotspots have been reported to correlate with nearby mitochondria (Spillane et al., 2013). We observed that Rab7a endosomes are frequently in contact with mitochondria in axons (Figures S5A and S5B). Live imaging showed that they often pause when they encounter mitochondria and form apparent contacts that persist for over 2 min (Figures S5C and S5E). Although close associations were also seen between Rab5a endosomes and mitochondria, these associations were brief and rarely persisted over prolonged periods (Figures S5D–S5F). In agreement with observations in HeLa cells (Wong et al., 2018), we found that contacts between constitutively active Rab7a (GFP-Rab7a^Q67L^) endosomes and mitochondria in axons remained for a longer time than those between wild-type Rab7a endosomes and mitochondria (Figures S5D–S5F).

To explore whether late endosomes in association with mitochondria coincide with translational hotspots, we first investigated the spatial relationship between RNA granules, mitochondria, and late endosomes (Figure 3G; Video S4). Approximately 35% of GFP-Rab7a endosomes were found adjacent to mitochondria, and 80% of these were associated with RNA granules (Figure 3H). We then asked whether newly synthesized proteins are found at these sites (Figures 3I and 3J). Indeed, 76% of Rab7a endosomes in close proximity to mitochondria had an enriched puromycin signal (Figure 3K), suggesting that late endosomes adjacent to mitochondria are sites of mRNA translation. We also noted that 44% of late endosomes with an enriched puromycin signal were not associated with mitochondria, indicating that not all endosome-associated translation occurs in the presence of mitochondria.

**Figure 4:**
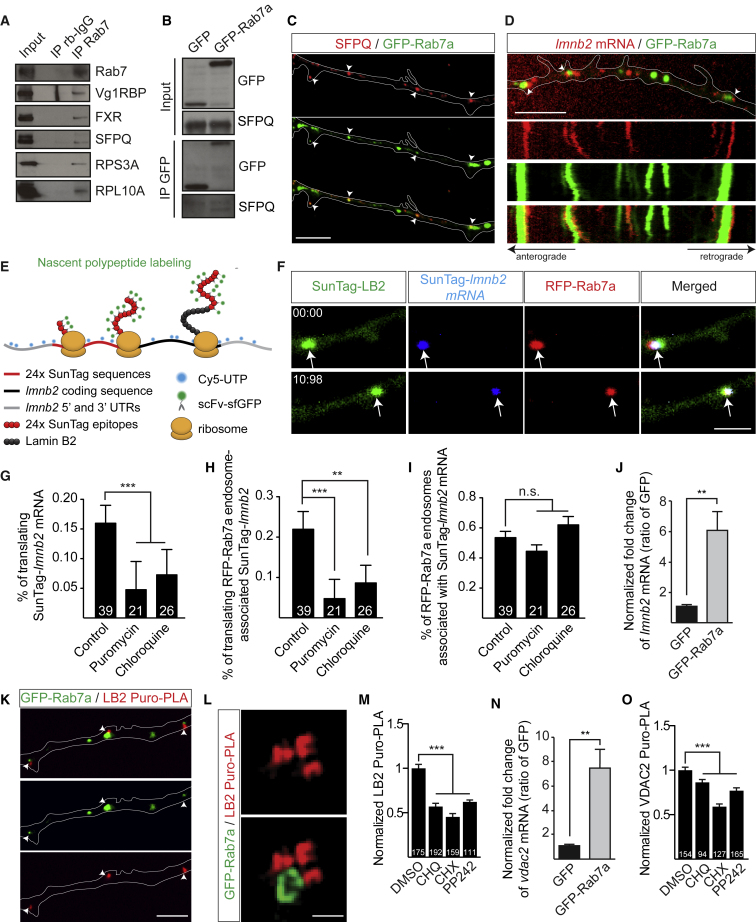
mRNAs Essential for Mitochondrial Integrity Are Translated on Rab7a Endosomes in Axons (A) Co-immunoprecipitation (coIP) between Rab7 and Vg1RBP, FXR, SFPQ, RPS3A, and RPL10A in brain lysates. (B) CoIP between GFP-Rab7a and SFPQ in brain lysates. (C) Colocalization of GFP-Rab7a with SFPQ in axons (white arrowheads). (D) RGC axon segment analyzed by live imaging and its corresponding kymograph (1 min), showing the association between Cy5-labeled *laminb2* mRNA (*lmnb2*) and GFP-Rab7a endosomes. (E) Schematic of SunTag system-based nascent polypeptide labeling to visualize Lamin B2 (LB2) synthesis. (F) Representative images showing the nascent SunTag-LB2 protein (green), the SunTag-*lmnb2* mRNA (blue), and the RFP-Rab7a endosome (red) in live axons (indicated by white arrows). (G) Percentage of SunTag-*lmnb2* mRNA associated with nascent SunTag-LB2 protein (translating) per 50-μm axon segment. (H) Percentage of SunTag-*lmnb2* mRNA associated with RFP-Rab7a endosomes colocalized with nascent SunTag-LB2 protein (translating). (I) Percentage of RFP-Rab7a endosomes associated with SunTag-*lmnb2* mRNA. (J) Quantitative analysis of *lmnb2* mRNA by qRT-PCR of cDNA synthesized from RNAs co-precipitated with GFP and GFP-Rab7a from brain extracts (n = 3 biological replicates). (K) Example of LB2 Puro-PLA signals in proximity to GFP-Rab7a endosomes (white arrowheads) in axons. (L) Super-resolution microscopy analysis of LB2 Puro-PLA signals next to a GFP-Rab7a endosome. (M) Quantification of LB2 Puro-PLA signals in treated RGC axons. (N) Quantitative analysis of *vdac2* mRNA by qRT-PCR of cDNA synthesized from RNAs co-precipitated with GFP and GFP-Rab7a from brain extracts (n = 3 biological replicates). (O) Quantification of VDAC2 Puro-PLA signals in treated RGC axons. N, number of axon segments in (G)–(I), (M), and (O). Mean ± SEM. ^∗^p < 0.05, ^∗∗^p < 0.01, ^∗∗∗^p < 0.001, Mann-Whitney test. Scale bars: 5 μm in (C), (D), and (K); 1 μm in (F); and 500 nm in (L). Time stamps are in the format of seconds:milliseconds. See also Video S4.

Video S4. Cy3-RNA Granules (Red) Associated with GFP-Rab7a Endosomes (Green) in Contact with Mitochondria (Blue), Related to Figure 4

### mRNAs Encoding Mitochondrial Proteins Are Translated on Rab7a Endosomes

The observed spatial coincidence of mitochondria, late endosomes, and nascent proteins raises the possibility that endosomes are sites for the translation of mRNAs encoding proteins that regulate or maintain mitochondrial function. To test this idea, we first focused on the RBP SFPQ (splicing factor proline- and glutamine-rich), which coordinates the axonal transport of mRNAs critical for mitochondria and is enriched near mitochondria and ribosomes in axons (Cosker et al., 2016). In addition to the previously identified RBPs, we found that SFPQ co-precipitated with endogenous Rab7 (Figure 4A) and with GFP-Rab7a (Figure 4B) in brain lysates. In axons, 47% of GFP-Rab7a endosomes colocalized with SFPQ (n = 137 vesicles in 80 axons) (Figure 4C).

Time-lapse imaging showed an association between Cy5-labeled *laminB2* mRNA (*lmnb2*), one of the SFPQ-regulated mRNAs important for mitochondrial integrity and axon survival (Cosker et al., 2016, Yoon et al., 2012), and GFP-Rab7a endosomes in axons (Figure 4D). To investigate whether the Rab7a endosome-associated *lmnb2* mRNA is translationally active, we used live single-molecule nascent polypeptide imaging based on the SunTag fluorescent tagging system to visualize axonal Lamin B2 (LB2) protein synthesis in real time (Figure 4E; Wang et al., 2016, Wu et al., 2016, Yan et al., 2016). As the nascent peptide is synthesized, super-folder GFP-tagged single-chain antibody fragments (scFv-sfGFP) rapidly bind to the 24 SunTag epitopes fused to the N-terminal of LB2, visible as a bright fluorescent spot. Cy5-labeled SunTag-*lmnb2* mRNA was electroporated into eye primordia of embryos expressing RFP-Rab7a and scFv-sfGFP. We observed a striking colocalization between the three signals: RFP-Rab7a endosomes, Cy5-SunTag-*lmnb2* mRNA, and sfGFP-labeled newly synthesized SunTag-LB2 proteins in axons (Figure 4F; Video S5). As expected, the proportion of actively translating mRNAs in axons or on late endosomes, indicated by the percentage of Cy5-SunTag-*lmnb2* colocalizing with SunTag-LB2 spots, was decreased by exposing the axons to a high concentration of puromycin, which blocks protein synthesis and induces the release of nascent polypeptides (Figures 4G and 4H). Interestingly, the percentage of Rab7a endosomes associated with SunTag-*lmnb2* mRNA remained unchanged (Figure 4I). The same effect was observed following application of CHQ (Figures 4G-I) suggesting that perturbing late endosome maturation impairs mRNA translation, not mRNA binding.

Video S5. Axonal Synthesis of LaminB2 (Green) on RFP-Rab7a Endosomes (Red), Associated with *laminb2* mRNA (Blue), Visualized with the SunTag Fluorescent Tagging System, Related to Figure 6

To see whether endogenous *lmnb2* mRNA could be observed on endosomes, we first used co-immunoprecipitation (coIP) followed by qRT-PCR and found that endogenous *lmnb2* mRNA was associated with Rab7a endosomes (Figure 4J). We then visualized the synthesis of native LB2 using puromycylation coupled with a proximity ligation assay (Puro-PLA) (tom Dieck et al., 2015) and found the LB2 Puro-PLA signal in close proximity to Rab7a endosomes (Figure 4K). This was further confirmed by super-resolution microscopy, which revealed LB2 Puro-PLA puncta decorating Rab7a endosomes (Figure 4L). Application of CHQ, CHX, and PP242 significantly reduced the amount of LB2 Puro-PLA puncta in axons (Figure 4M), indicative of protein synthesis inhibition. To extend these findings to another mitochondrion-related protein, we examined the axonally synthesized voltage-dependent anion-selective channel protein 2 (VDAC2) (Shigeoka et al., 2016), which is involved in exchanging solutes across the outer mitochondrial membrane (Naghdi and Hajnóczky, 2016). We found that *vdac2* mRNA also co-precipitated with GFP-Rab7a (Figure 4N). We then confirmed that VDAC2 was locally synthesized by Puro-PLA and that CHQ, CHX, and PP242 reduced the signal in axons (Figure 4O). Taken together, these results show that late endosome-coupled translation can supply nascent proteins that have a role in maintaining axonal mitochondrial function.

### CMT2B-Associated Rab7a Mutations Cause Dominant Effects on Late Endosomal Trafficking and Axonal Integrity

**Figure 5:**
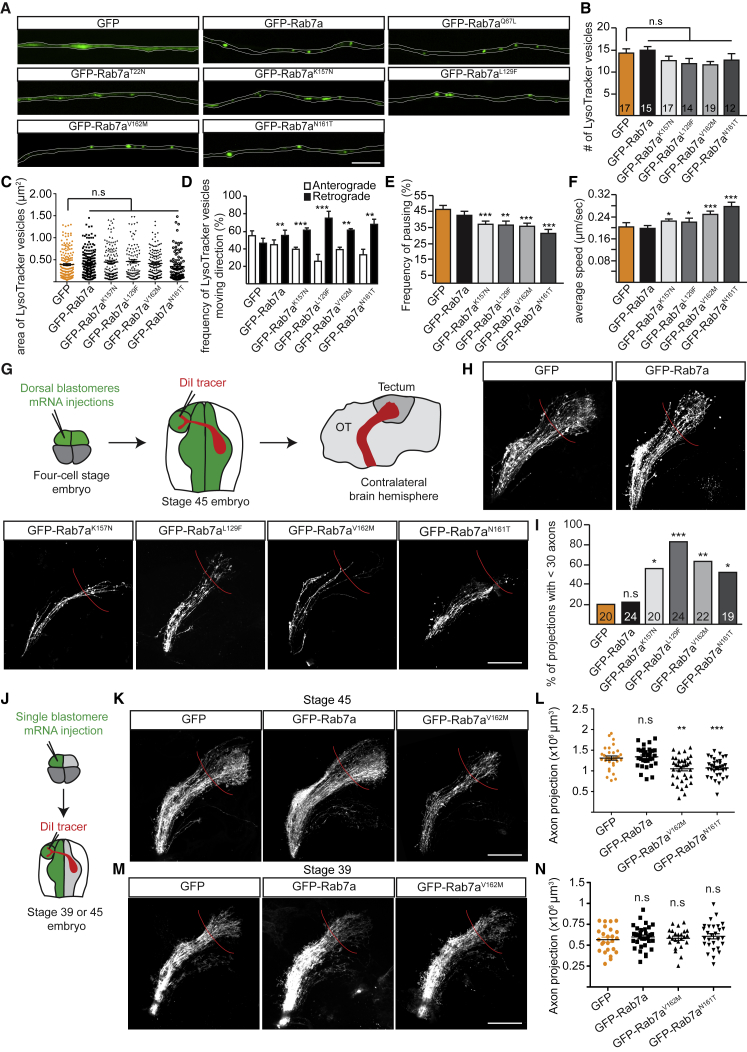
Expression of Rab7a CMT2B Mutants Perturbs Late Endosome Trafficking and Axonal Projection *In Vivo* (A) Representative images of wild-type or mutant GFP-Rab7a endosomes in cultured axons. (B) Number of LysoTracker-positive vesicles per 50 μm of axon (n, number of axon segments). (C) Area of individual LysoTracker vesicles in axons (n = 180 [GFP], 187 [GFP-Rab7a], 137 [GFP-Rab7a^K157N^], 97 [GFP-Rab7a^L129F^], 140 [GFP-Rab7a^V162M^], 89 [GFP-Rab7a^N161T^] vesicles). (D) Percentage of LysoTracker vesicles moving anterogradely or retrogradely over a 1-min video (n = 130 [GFP], 114 [GFP-Rab7a], 249 [GFP-Rab7a^K157N^], 78 [GFP-Rab7a^L129F^], 155 [GFP-Rab7a^V162M^], 96 [GFP-Rab7a^N161T^] vesicles). (E and F) Frequency of pausing (E) and average speed (F) of individual vesicles over a 1-min video (n = 130 [GFP], 114 [GFP-Rab7a], 249 [GFP-Rab7a^K157N^], 78 [GFP-Rab7a^L129F^], 155 [GFP-Rab7a^V162M^], 96 [GFP-Rab7a^N161T^] vesicles). (G) Schematic of labeling of stage 45 RGC axons *in vivo* in bilaterally injected embryos. OT, optic tract. (H) Representative images of RGC axon projections in stage 45 embryos expressing GFP, wild-type Rab7a, or CMT2B disease mutants. (I) Percentage of embryos with defective RGC projections with less than 30 axons. (J) Schematic of RGC axon labeling in unilaterally injected embryos. (K) Representative images of RGC axon projections in stage 45 embryos expressing GFP, wild-type Rab7a, or GFP-Rab7a^V162M^. (L) Axon projection volume post-optic chiasm in stage 45 embryos expressing GFP (n = 31 embryos), wild-type Rab7a (n = 32 embryos), GFP-Rab7a^V162M^ (n = 37 embryos), or GFP-Rab7a^N161T^ (n = 33 embryos). (M) Representative images of RGC axon projections in stage 39 embryos expressing GFP, wild-type Rab7a, or GFP-Rab7a^V162M^. (N) Axon projection volume post-optic chiasm in stage 39 embryos expressing GFP (n = 25 embryos), wild-type Rab7a (n = 33 embryos), GFP-Rab7a^V162M^ (n = 24 embryos), or GFP-Rab7a^N161T^ (n = 28 embryos). Mean ± SEM. ^∗^p < 0.05, ^∗∗^p < 0.01, ^∗∗∗^p < 0.001; Fisher’s exact test in (D) or Mann-Whitney test in (E), (F), (I), (L), and (N). Scale bars, 10 μm in (A) and 100 μm in (H), (K), and (M).

The autosomal-dominant neurological disorder CMT2B is associated with missense mutations of four amino acids in the human *rab7a* gene (Cogli et al., 2009). This rare neuropathy primarily affects the peripheral sensorimotor systems, leading to distal sensory loss, muscle weakness, and atrophy. The mechanism behind how these mutations induce axonopathy remains elusive (BasuRay et al., 2010, Cherry et al., 2013, Cogli et al., 2010, Janssens et al., 2014, Liu and Wu, 2017, Spinosa et al., 2008). We wondered whether our findings of late endosome-associated axonal translation, especially of mRNAs essential for mitochondrial and axonal integrity, could be relevant to understanding CMT2B. Because the amino acid sequence of *Xenopus* Rab7a is 96% identical to human Rab7a, including the four residues identified as mutated in CMT2B patients (L129F, K157N, N161T/I, and V162M), we generated four GFP-tagged pathological mutants (Rab7a^K157N^, Rab7a^L129F^, Rab7a^V162M^, and Rab7a^N161T^) and expressed them in the *Xenopus* RGCs. In distal RGC axons expressing CMT2B-linked mutants, discrete GFP-positive puncta were clearly visible with each mutant, resembling patterns observed in wild-type GFP-Rab7a and CA GFP-Rab7a^Q67L^, in contrast to the more diffuse signal observed for the DN GFP-Rab7a^T22N^ (Figure 5A). Live imaging revealed that expression of CMT2B mutants affects the direction, frequency of pausing, and average speed of LysoTracker-positive vesicles in RGC axons (Figures 5B–5F), suggesting conserved dominant effects of these mutants on late endosomal trafficking in our system (Ponomareva et al., 2016, Zhang et al., 2013).

Severe defects in RGC axonal projections *in vivo* were also noted in DiI labeling experiments at stage 45, when most of these axons have established synaptic connections in the optic tectum of the midbrain. The majority of embryos expressing Rab7a disease mutants had fewer axons in the optic tract and the optic tectum (Figures 5G–5I). To test whether this effect on axons was autonomous, we performed mRNA injections into only one of the two dorsal blastomeres at the four-cell stage, leading to embryos in which expression was restricted to one-half of the CNS (Figure 5J). As RGC axons in *Xenopus* cross at the optic chiasm, contralaterally projecting mutant axons navigate through wild-type tissue. Although less severe, RGCs axons expressing GFP-Rab7a^V162M^ or GFP-Rab7a^N161T^ in the wild-type brain also displayed less dense projections at stage 45 (Figures 5K and 5L). To distinguish whether the defects at stage 45 optic projections were the result of a failure to grow initially or degeneration after growth, we examined the projections 3–4 days earlier, at stage 39. No obvious axon projection phenotypes were observed in mutant-expressing embryos at stage 39 (Figures 5M and 5N), suggesting that Rab7a-CMT2B mutants affect axonal maintenance after their initial growth.

### CMT2B-Associated Rab7a Mutations Disrupt Axonal Translation of mRNAs Essential for Mitochondrial Integrity

**Figure 6:**
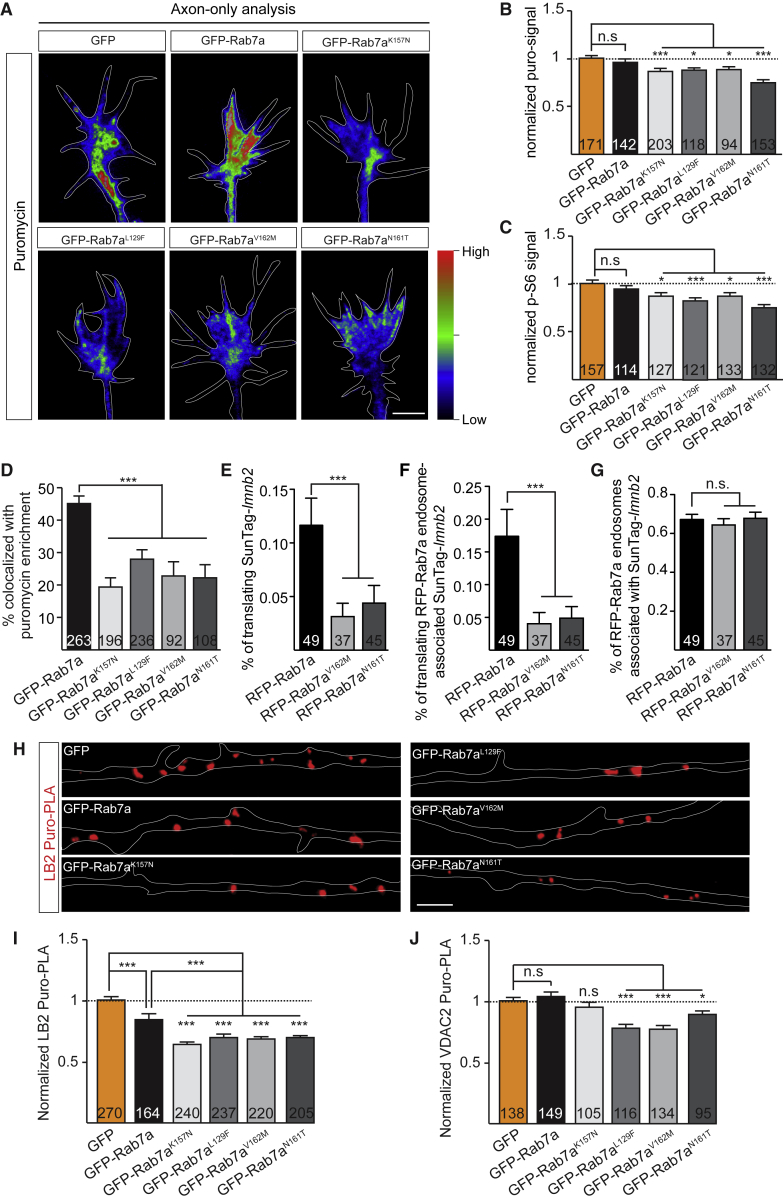
CMT2B-Associated Rab7a Mutations Disrupt Axonal Translation of mRNAs Essential for Mitochondrial Integrity (A) Heatmaps indicating relative puromycin fluorescence in RGC growth cones. (B) QIF analysis of puromycin incorporation (puro-signal) in somaless RGC growth cones expressing the indicated constructs. (C) QIF analysis of phospho-S6 ribosomal proteins in growth cones expressing the indicated constructs. (D) Percentage of GFP-Rab7a CMT2B mutant-endosomes with puromycin enrichment in axon shafts are decreased compared with wild-type GFP-Rab7a endosomes. (E) Percentage of SunTag-*lmnb2* mRNA associated with nascent SunTag-LB2 protein (translating) per 50-μm axon segment. (F) Percentage of SunTag-*lmnb2* mRNA associated with RFP-Rab7a or Rab7a CMT2B mutant endosomes colocalized with nascent SunTag-LB2 protein (translating). (G) Percentage of RFP-tagged Rab7a or Rab7a CMT2B mutant endosomes colocalized with Cy5-labeled SunTag-*lmnb2* mRNA. (H) Representative LB2 Puro-PLA signals in axons expressing GFP, GFP-Rab7a, and GFP-Rab7a CMT2B mutants. (I) Quantification of LB2 Puro-PLA signals in axons expressing GFP, GFP-Rab7a, and GFP-Rab7a CMT2B mutants. (J) Quantification of VDAC2 Puro-PLA signals in axons expressing GFP, GFP-Rab7a, and GFP-Rab7a CMT2B mutants. Mean ± SEM. N, number of growth cones in (B) and (C), number of endosomes in (D), or number of axon segments in (E–G), (I), and (J). ^∗^p < 0.05, ^∗∗∗^p < 0.001, Mann-Whitney test. Scale bars, 5 μm in (A) and (H). See also Video S5.

The loss of axonal integrity induced by CMT2B mutants could be preceded by impaired intra-axonal translation. Indeed, puromycin labeling revealed a decrease in nascent protein synthesis in growth cones of somaless RGC axons from stage 33/34 embryos expressing each of the four CMT2B mutants compared with GFP-Rab7a or the GFP control (Figures 6A and 6B). The translation marker p-S6 in growth cones was also reduced by all four of the CMT2B-related Rab7a mutants (Figure 6C). Finally, the percentage of Rab7a CMT2B mutant endosomal puncta colocalizing with enriched puromycin signal was significantly decreased compared with wild-type GFP-Rab7a endosomes (Figure 6D).

To test whether the synthesis of proteins needed for mitochondrial function was affected in axons expressing CMT2B mutants, we first used the SunTag method and found that the proportion of actively translating SunTag-*lmnb2* mRNAs was significantly reduced in RFP-Rab7a^V162M^- or RFP-Rab7a^N161T^-expressing axons (Figure 6E). Moreover, there was a significant reduction in newly synthesized SunTag-LB2 proteins on mutant-associated endosomes compared with wild-type RFP-Rab7a endosomes (Figure 6F). Interestingly, the percentage of wild-type or mutant endosomes associated with Cy5-labeled mRNA remained constant (Figure 6G), suggesting that Rab7a disease mutations do not alter the association of *lmnb2* mRNAs with late endosomes but, rather, diminish their translation.

Next, we asked whether the synthesis of native LB2 and VDAC2 proteins was affected by expressing CMT2B-related Rab7a mutants. Overexpression of GFP-Rab7a led to an approximately 20% decrease in nascent axonal LB2, as measured by LB2 Puro-PLA (Figures 6H and 6I), suggesting that LB2 local synthesis is sensitive to altered Rab7a levels in axons. Expression of each of the four Rab7a CMT2B mutants, however, caused a significantly larger drop (an approximately 35% decrease) in new LB2 synthesis (Figures 6H and 6I). Three of four mutants, but not wild-type GFP-Rab7a, resulted in a reduction in VDAC2 Puro-PLA puncta (Figure 6J). Together, these results suggest that CMT2B Rab7a mutations compromise axonal translation of mRNAs, including those encoding proteins essential for mitochondrial integrity.

### CMT2B-Associated Rab7a Mutations Disrupt Mitochondrial Integrity

**Figure 7:**
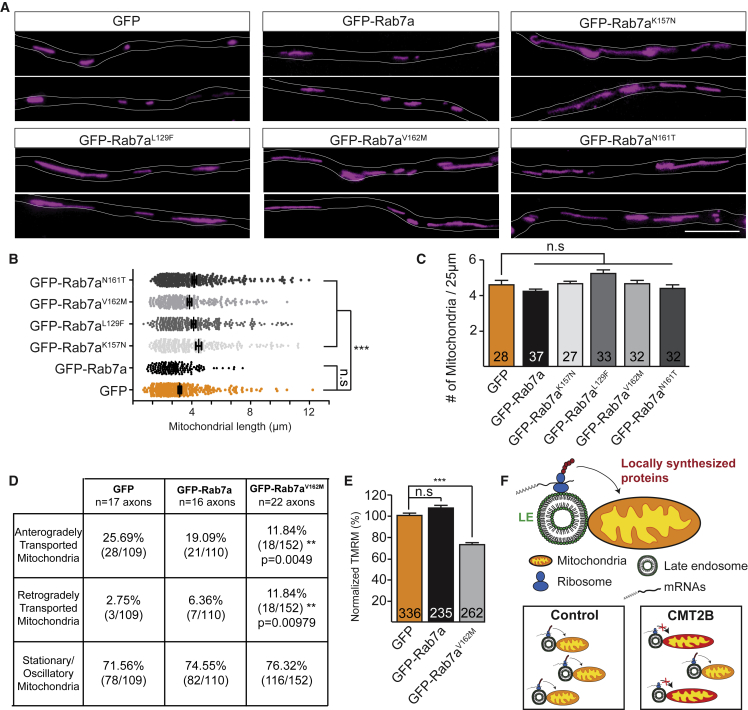
Expression of CMT2B-Associated Rab7a Mutations Compromises Mitochondrial Integrity (A) MitoTracker-labeled mitochondria illustrating variation in mitochondrial morphology in axons expressing GFP-Rab7a CMT2B mutants. (B) Quantification of mitochondrial length (n = 301 [GFP], 148 [GFP-Rab7a], 273 [GFP-Rab7a^K157N^], 268 [GFP-Rab7a^L129F^], 225 [GFP-Rab7a^V162M^], 279 [GFP-Rab7a^N161T^] vesicles). (C) Quantification of the number of mitochondria within 25-μm axon segments. (D) The percentage of mitochondria transported anterogradely decreases in axons expressing the GFP-Rab7a^V162M^ CMT2B mutant, whereas the percentage of mitochondria transported retrogradely increases compared with the GFP control or GFP-Rab7a-expressing axons. (E) Quantification of mitochondrial membrane potential, measured by TMRM fluorescence intensity, showing a decrease in axons expressing the GFP-Rab7a^V162M^ CMT2B mutant (n = 31 axons) compared with the GFP control (n = 44 axons) and GFP-Rab7a-expressing axons (n = 31 axons). (F) Proposed model. mRNAs and translation machinery associate with late endosomes in axons. Late endosomes in proximity to mitochondria are sites for synthesis of proteins essential for mitochondrial integrity. Late endosome-sited translation is defective in axons expressing CMT2B-associated Rab7a mutants, causing a reduction in mitochondrion-related mRNAs translation. N, number of axon segments in (C); N, number of mitochondria in (E). Mean ± SEM. ^∗∗∗^p < 0.001, Mann-Whitney test. Scale bars, 5 μm in (A).

Because CMT2B-associated Rab7a mutants downregulate axonal translation of mitochondrial proteins, we wondered whether mutant-expressing axons exhibit abnormal mitochondrial phenotypes. To address this question, we first analyzed mitochondrial morphology in RGC axons expressing each of the four CMT2B Rab7a mutants. MitoTracker-labeled mitochondria in CMT2B Rab7a mutant-expressing axons exhibited highly elongated profiles (Figure 7A) compared with GFP-Rab7a or GFP controls (Figure 7B), whereas the density of mitochondria remained unchanged (Figure 7C), consistent with the mitochondrial phenotypes seen when axonal LB2 synthesis was inhibited (Yoon et al., 2012). Defective axonal mitochondrial trafficking is a sign of altered mitochondrial physiology (Roque et al., 2016, Sheng and Cai, 2012), so we analyzed mitochondrial dynamics in axons expressing Rab7a^V162M^ during 5-min time-lapse imaging intervals. We found a decrease in anterograde mitochondrial transport in CMT2B mutant-expressing axons (Figure 7D) and an increase in retrograde transport (Figure 7D) compared with GFP-Rab7a or GFP control axons. Finally, we measured the mitochondrial membrane potential (ΔΨm), which drives ATP production, by quantifying the accumulation of the cationic fluorescent probe tetramethylrhodamine methyl ester (TMRM) in mitochondria along axons and found a significant ΔΨm reduction in axons expressing Rab7a^V162M^ compared with the GFP control or wild-type GFP-Rab7a (Figure 7E).

## Discussion

Our results support a model in which Rab7a endosomes are sites for mRNA translation in axons, generating focal hotspots of local protein synthesis often associated with mitochondria (Figure 7F). Moreover, we found that translation of nucleus-encoded mitochondrial mRNAs essential for mitochondrial function occurs at these hotspots.

Axonal mRNA localization relies on specific association with RBPs and targeted transport through direct or indirect binding with cytoskeletal motor proteins (Buxbaum et al., 2015). Our live-imaging analyses of RNA granules, mRNAs, and RBPs collectively support this view because they provide evidence for directed transport of RNPs in RGC axons, mostly independent of endosomal trafficking. However, we also found that around a quarter of oscillatory and slow-moving RNPs are associated with endosomes in axons. How RNPs become tethered to the endosomes remains to be determined. A lipid-binding adaptor protein has been proposed as the mechanism for mRNA recruitment on endosomes in fungal hyphae (Pohlmann et al., 2015). This mechanism may be conserved because several different RBPs (SFPQ, Vg1RBP, and FXR) were found to be part of the endosome-associated complex in our system.

It has been suggested that ribosomes assemble on endosome-associated mRNAs to activate translation (Higuchi et al., 2014), and we found ribosomal proteins associated with late endosomes in axons. In addition, the enrichment of newly synthesized proteins at Rab7a endosomes indicates that late endosomes are hotspots of local protein synthesis. Rab7a function is required for axonal translation because both CA and DN Rab7a mutants caused downregulation of translation. Previous work has shown that the endosomal sorting complex required for transport II (ESCRT-II) colocalizes with Rab7 in RGC axons, and ESCRT-II knockdown causes a similar decrease (∼20%) in RGC axonal protein synthesis (Konopacki et al., 2016), suggesting a potential role of ESCRT-II in this process. Not all intra-axonal translation requires endosomal function; the protein synthesis inhibitor CHX decreases axonal protein synthesis more severely (∼50%). Our results also showed the presence of mRNAs and ribosomes on early Rab5a endosomes, but neither expression of Rab5a mutants nor the endocytosis inhibitor dynasore revealed significant effects on constitutive axonal protein synthesis. Future work will be needed to determine why late but not early endosomes seem to regulate axonal translation in this system.

Translation hotspots have previously been reported in both axons and dendrites (Eberwine et al., 2001, Kim et al., 2013, Spillane et al., 2013, Wong et al., 2017). In axons, hotspots correlate with the presence of mitochondria (Spillane et al., 2013). In accordance with these observations, we found that hotspots of late endosome-associated translation were often in close proximity to mitochondria. Strikingly, live imaging revealed that Rab7a endosomes remained in contact with mitochondria for prolonged periods. A direct association between late endosomes or lysosomes and mitochondria has been reported in HeLa cells and correlates with mitochondrial fission events (Wong et al., 2018). However, no mitochondrial fission event was observed in RGC axons when late endosomes docked on mitochondria, suggesting that other molecular activities may also be involved in these interactions. Mitochondrion-linked mRNAs represent one of the main categories of translating mRNAs in adult axon terminals in the mouse (Shigeoka et al., 2016), and their local translation is essential for the maintenance of mitochondrial function (Gale et al., 2018, Kaplan et al., 2009, Yoon et al., 2012). We found that late endosomes are sites of axonal translation of mRNAs that are essential for mitochondria. Indeed, the RBP SFPQ, which orchestrates a mitochondrion-related RNA regulon (Cosker et al., 2016), and its cargo *lmnb2* mRNA were found on late endosomes in axons. Moreover, we showed that Rab7a endosomes are sites for the local synthesis of LB2, which is key to mitochondrial and axonal integrity (Cosker et al., 2016, Yoon et al., 2012). It is not yet clear whether the association with mitochondria can stimulate late endosomal-sited translation, and identification of a tethering complex may be essential to answer this question. In addition, the observed close association of the ER with RNA granules and late endosomes in RGC axons opens the possibility of its involvement in late endosomal trafficking and/or translation-associated activity. It is also of interest to note that, although mitochondrial mRNAs represent only 5% of the total RGC axonal translatome (Shigeoka et al., 2016), global axonal protein synthesis decreases by 20% when Rab7a function or late endosome maturation is perturbed. This suggests that endosome-associated translation is involved in other local physiological processes in axons, and our finding that *β-actin* mRNAs also associate with endosomes supports this idea.

The inherited peripheral neuropathy CMT2B is caused by any one of the five *rab7a* missense mutations (e.g., L129F, K157N, N161T/I, and V162M) (Houlden et al., 2004, Meggouh et al., 2006, Verhoeven et al., 2003, Wang et al., 2014). Despite Rab7a being a ubiquitous protein, axons of peripheral neurons are particularly susceptible to CMT2B-associated mutations. Different molecular mechanisms have been proposed to explain this. *rab7a* CMT2B-associated mutations show perturbed axonal transport of late endosomes (Ponomareva et al., 2016, Zhang et al., 2013), an effect we also observed in our system. These disrupted endosomal dynamics are correlated with altered signaling responses to neurotrophic factors in axons, in particular nerve growth factor (NGF) (BasuRay et al., 2010, Zhang et al., 2013). Another explanation is that *rab7a* mutations disrupt the regulation of effectors that function specifically in the peripheral nervous system, such as the intermediate filament peripherin, which has been shown to interact directly with Rab7a (Cogli et al., 2013). In this study, we propose an additional mechanism that may contribute to CMT2B disease; i.e., that Rab7a mutants affect local translation. Local translation is thought to be particularly important in the distal portions of long axons, such as sensory and motor neurons, because it supplies new proteins to meet local demand far from the soma. In this regard, it is of note that the axonal phenotypes observed in CMT2B-expressing peripheral sensory axons (Ponomareva et al., 2016), and retinotectal axons in this study, are typical of the axonal phenotypes seen when subcellular RNA-based mechanisms are dysregulated (Yoon et al., 2012). We found compromised local protein synthesis in CMT2B mutant-expressing axons and, in particular, of known axonal survival factors, such as LB2. The fact that CMT2B mutants did not affect the association of *lmnb2* mRNAs with late endosomes but did reduce protein synthesis suggests that these Rab7a disease mutants affect the efficiency of endosome-sited translation. Further work will be required to understand the exact mechanistic role of Rab7a in this process and how much the reduced local translation contributes to the axonal and mitochondrial phenotypes seen in CMT2B mutant-expressing axons. In view of the emerging functional interactions between endosomes and mitochondria, it will also be of interest in the future to find out whether other aspects of the cross-talk between these two organelles are modulated by Rab7a functions in axons and whether these are perturbed in CMT2B disease.

## STAR★Methods

### Key Resources Table

**Table:** 

REAGENT or RESOURCE	SOURCE	IDENTIFIER
**Antibodies**		

Rabbit polyclonal anti-RPS3A	Proteintech	Cat#14123-1-AP; RRID:AB_2253921
Mouse polyclonal anti-RPS3A	Abcam	Cat#ab194670; RRID:AB_2756396
Mouse polyclonal anti-RPL19	Abcam	Cat#ab58328; RRID:AB_945305
Rabbit polyclonal anti-RPL24	Proteintech	Cat#17082-1-AP; RRID:AB_2181728
Rabbit polyclonal anti-Vg1RBP	Gift from Dr Nancy Standart, Cambridge	N/A
Rabbit polyclonal anti-LAMP-1	Abcam	Cat#ab24170; RRID:AB_775978
Mouse monoclonal anti-FXR	Gift from Dr Edward Khandjian, University of Quebec	N/A
Mouse monoclonal anti-Rab5	Santa Cruz	Cat#sc-46692; RRID:AB_628191
Rabbit monoclonal anti-Rab7	Abcam	Cat#ab137029; RRID:AB_2629474
Rabbit polyclonal anti-Rab7	Abcam	Cat#ab77993; RRID:1566661
Mouse monoclonal anti-GFP	Abcam	Cat#ab1218; RRID:AB_298911
Rabbit IgG, polyclonal, Isotype control	Abcam	Cat#ab37415; RRID:AB_2631996
Mouse monoclonal anti-GM130	BD Biosciences	Cat#BD610822; RRID:AB_2756397
Rabbit polyclonal anti-RPL10A	Proteintech	Cat#16681-1-AP; RRID:AB_2181281
Rabbit polyclonal anti-phospho-AKT (Ser473)	Cell Signaling	Cat#9271T; RRID:AB_329825
Rabbit monoclonal anti-phospho-mTOR (Ser2448)	Abcam	Cat#ab109268; RRID:AB_10888105
Rabbit monoclonal anti-mTOR	Cell Signaling	Cat#2983; RRID:AB_2105622
Rabbit polyclonal anti-phospho-S6 Ribosomal Protein (Ser235/236)	Cell Signaling	Cat#2211; RRID:AB_331679
Mouse monoclonal anti-puromycin, clone 12D10	Sigma-Aldrich	Cat#MABE343; RRID:AB_2566826
Mouse monoclonal anti-puromycin, clone 12D10, Alexa Fluor 647 Conjugate	Sigma-Aldrich	Cat#MABE343-AF647; RRID:AB_2736876
Rabbit polyclonal anti-SFPQ	Abcam	Cat#ab38148; RRID:AB_945424
Rabbit polyclonal anti-Lamin B2	Abcam	Cat#ab97513; RRID:AB_10681013
Goat polyclonal anti-VDAC2	Abcam	Cat#ab37985; RRID:AB_778790
Goat anti-mouse Alexa Fluor 488	ThermoFisher Scientific	Cat#A-11001; RRID:AB_2534069
Goat anti-mouse Alexa Fluor 568	ThermoFisher Scientific	Cat#A-11004; RRID:AB_141371
Goat anti-rabbit Alexa Fluor 488	ThermoFisher Scientific	Cat#A-11008; RRID:AB_143165
Goat anti-rabbit Alexa Fluor 568	ThermoFisher Scientific	Cat#A-11011; RRID:AB_143157

**Bacterial and Virus Strains**		

BioBlue Chemically Competent Cells	Bioline	Cat#BIO-85037

**Chemicals, Peptides, and Recombinant Proteins**		

Leibovitz’s L-15 Medium	ThermoFisher	Cat#11415064
Antibiotic-Antimycotic (100X)	ThermoFisher	Cat#15240062
Poly-L-lysine	Sigma-Aldrich	Cat#P1274
Laminin	Sigma-Aldrich	Cat#L2020
Cy3-UTP	PerkinElmer	Cat#NEL582001EA
Cy5-UTP	PerkinElmer	Cat#NEL583001EA
Puromycin	Sigma-Aldrich	Cat#P8833
CHX	Sigma-Aldrich	Cat#C4859
Dynasore	Sigma-Aldrich	Cat#D7693
Chloroquine	Sigma-Aldrich	Cat#C6628
PP242	Tocris	Cat#4257
SUPERase In RNase Inhibitor	Ambion	Cat#AM2696
MitoTracker Deep Red FM	ThermoFisher Scientific	Cat#M22426
LysoTracker Red CMXRos	ThermoFisher Scientific	Cat#M7512
ER-Tracker Green	ThermoFisher Scientific	Cat#E34251
1,1’-Dioctadecyl-3,3,3′,3′-Tetramethylindocarbocyanine Perchlorate (Dil)	ThermoFisher Scientific	Cat#D282
Tetramethylrhodamine, methyl ester (TMRM)	ThermoFisher Scientific	Cat#T668
FluorSave	Merck Millipore (Calbiochem)	Cat#345789-20
Vectashield mounting medium with DAPI	Vector Laboratories	Cat#H-1200

**Critical Commercial Assays**		

SuperScript III First-Strand Synthesis System	ThermoFisher Scientific	Cat#18080051
QuantiTect SYBR Green PCR kit	QIAGEN	Cat#204141
QuikChange II Site-Directed Mutagenesis Kit	Agilent Technologies	Cat#200555
mMessage mMachine SP6 Transcription Kit	ThermoFisher Scientific	Cat#AM1340
Poly(A) Tailing Kit	ThermoFisher Scientific	Cat#AM1350
RNeasy Mini Kit	QIAGEN	Cat#74104
Duolink *In Situ* PLA Probe Anti-Rabbit PLUS Affinity purified Donkey anti- Rabbit IgG	Sigma-Aldrich	Cat#DUO92002
Duolink *In Situ* PLA Probe Anti-Goat PLUS Affinity purified Donkey anti- Goat IgG	Sigma-Aldrich	Cat#DUO92003
Duolink *In Situ* PLA Probe Anti-Mouse MINUS Affinity purified Donkey anti-Mouse IgG	Sigma-Aldrich	Cat#DUO92004
Duolink *In Situ* Detection Reagents Red	Sigma-Aldrich	Cat#DUO92008
Dynabeads Antibody Coupling kit	Invitrogen	Cat#14311D

**Experimental Models: Organisms/Strains**		

*Xenopus laevis*	Nasco	Cat#LM00715 (male); RRID:XEP_Xla100, Cat#LM00535 (female); RRID: XEP_Xla

**Oligonucleotides**		

Molecular beacon: MB1 Cy3-oCoGoAoCoGoCoU+CoA oGoUoU+AoGoG+AoUoUoUoUoC+AoUoGoCoGoUo CoG-BHQ2	(Turner-Bridger et al., 2018)	N/A
Molecular beacon: MB2 Cy3-oGoCoGoCoAoG+GoAoA+ GoCoCoAoA+GoAoUoG+GoAoUoGoCoGoC-BHQ2	(Turner-Bridger et al., 2018)	N/A
Primers for *actb* (*Xenopus laevis*) qRT-PCR forward: 5′-CCAGAAGAACACCCAGTGCT-3′	This study	N/A
Primers for *actb* (*Xenopus laevis*) qRT-PCR reverse: 5′-CAGGGACAACACAGCTTGGA-3′	This study	N/A
Primers for *lmnb2* (*Xenopus laevis*) qRT-PCR forward: 5′-GCAAGTGAAGATGTACAAGGAAGAA-3′	This study	N/A
Primers for *lmnb2* (*Xenopus laevis*) qRT-PCR reverse: 5′-CGTCGCTCAGTTAATTCTTCTAGGG-3′	This study	N/A
Primers for *vdac2* (*Xenopus laevis*) qRT-PCR forward: 5′-ACTCGCATGCAGCCATATCT-3′	This study	N/A
Primers for *vdac2* (*Xenopus laevis*) qRT-PCR reverse: 5′-TACCAGCGCAGAAAGTGTGA-3′	This study	N/A

**Recombinant DNA**		

Plasmid: pCS2+-GFP	(Das et al., 2003)	N/A
Plasmid: pCS2+-GFP-Rab5a (*Xenopus laevis*)	(Falk et al., 2014)	N/A
Plasmid: pCS2+-RFP-Rab5a (*Xenopus laevis*)	(Falk et al., 2014)	N/A
Plasmid: pCS2+-GFP-Rab7a (*Xenopus laevis*)	(Falk et al., 2014)	N/A
Plasmid: pCS2+-RFP-Rab7a (*Xenopus laevis*)	(Falk et al., 2014)	N/A
Plasmid: pCS2+-GFP-Rab5a^Q80L^ (*Xenopus laevis*)	This study	N/A
Plasmid: pCS2+-GFP-Rab5a^S35N^ (*Xenopus laevis*)	This study	N/A
Plasmid: pCS2+-GFP-Rab7a^Q67L^ (*Xenopus laevis*)	(Falk et al., 2014)	N/A
Plasmid: pCS2+-GFP-Rab7a^T22N^ (*Xenopus laevis*)	(Falk et al., 2014)	N/A
Plasmid: pCS2+-GFP-Rab7a^L129F^ (*Xenopus laevis*)	This study	N/A
Plasmid: pCS2+-GFP-Rab7a^K157N^ (*Xenopus laevis*)	This study	N/A
Plasmid: pCS2+-GFP-Rab7a^N161T^ (*Xenopus laevis*)	This study	N/A
Plasmid: pCS2+-GFP-Rab7a^V162M^ (*Xenopus laevis*)	This study	N/A
Plasmid: pCS2+-RFP-Rab7a^N161T^ (*Xenopus laevis*)	This study	N/A
Plasmid: pCS2+-RFP-Rab7a^V162M^ (*Xenopus laevis*)	This study	N/A
Plasmid: pCS2+-Lamp1-RFP	(Sherer et al., 2003)	Re-cloned from Addgene Cat#1817; RRID:Addgene_1817
Plasmid: pCS2+-Rps3a-GFP (*Xenopus laevis*)	This study	N/A
Plasmid: pCS2+-Rps4x-GFP (*Xenopus laevis*)	This study	N/A
Plasmid: pCS2+-GFP-Vg1RBP	(Leung et al., 2006)	N/A
Plasmid: Mito-GFP	Gift from Dr Michael Coleman, Cambridge	N/A
Plasmid: pCS2+-*lmnb2* 5′UTR-24xSunTag-*lmnb2* CDS-*lmnb2*	(Klein et al., 2002, Yan et al., 2016)	Re-cloned from Source Bioscience SB5157193 (*lmnb2*); Addgene Cat#74928 (24xSunTag); RRID:Addgene_74928
Plasmid: pCS2+-scFv-sfGFP	(Tanenbaum et al., 2014)	Re-cloned from Addgene Cat#60907; RRID:Addgene_60907

**Software and Algorithms**		

Volocity	PerkinElmer	Version 6.0.1, RRID:SCR_002668
FIJI	(Schindelin et al., 2012)	Version 2.0.0-rc-65/1.51w, RRID:SCR_002285
MATLAB	MathWorks	Version R2016b, RRID:SCR_001622
GraphPad Prism	GraphPad	Version 5, RRID:SCR_002798

### Contact for Reagent and Resource Sharing

Further information and requests for resources and reagents should be directed to and will be fulfilled by the Lead Contact, Christine E. Holt (ceh33@cam.ac.uk).

### Experimental Model and Subject Details

#### *Xenopus laevis* Embryos

*Xenopus laevis* eggs were fertilized *in vitro* and embryos were raised in 0.1x Modified Barth’s Saline (MBS; 8.8mM NaCl, 0.1 mM KCl, 0.24mM NaHCO_3_, 0.1 mM HEPES, 82μM MgSO_4_, 33μM Ca(NO_3_)_2_, 41μM CaCl_2_) at 14-20°C and staged according to the tables of Nieuwkoop and Faber (Nieuwkoop and Faber, 1994). All animal experiments were approved by the University of Cambridge Ethical Review Committee in compliance with the University of Cambridge Animal Welfare Policy. This research has been regulated under the Animals (Scientific Procedures) Act 1986 Amendment Regulations 2012 following ethical review by the University of Cambridge Animal Welfare and Ethical Review Body (AWERB).

#### Primary *Xenopus* Retinal Cultures

Eye primordia were dissected from Tricaine Methanesulfonate (MS222) (Sigma-Aldrich) anesthetized embryos of either sex at stage 35/36 and cultured on 10μg/ml poly-L-lysine (Sigma-Aldrich)- and 10μg/ml laminin (Sigma-Aldrich)-coated glass bottom dishes (MatTek) in 60% L-15 medium (ThermoFisher), 1x Antibiotic-Antimycotic (ThermoFisher) at 20°C for 24-48 hours. 10-20 eye primordia (from 5-10 embryos) were cultured per dish and, typically, 2-3 dishes were used per experimental condition for each biological replicate.

### Method Details

#### Constructs

GFP- or RFP-tagged *Xenopus* Rab constructs GFP-Rab5a, RFP-Rab5a, GFP-Rab7a, RFP-Rab7a, constitutively active mutant GFP-Rab7a^Q67L^, dominant negative mutant GFP-Rab7a^T22N^ in pCS2+ vector were previously reported (Falk et al., 2014). Point mutations of GFP-Rab5a to obtain the constitutively active mutant GFP-Rab5a^Q80L^ and dominant negative mutant GFP-Rab5a^S35N^, and GFP-Rab7a or RFP-Rab7a to obtain the four CMT2B-associated mutants Rab7a^K157N^, Rab7a^L129F^, Rab7a^V162M^ and Rab7a^N161T^ were performed by site-directed mutagenesis using QuikChange II Site-Directed Mutagenesis Kit (Agilent Technologies). Human and *Xenopus* Rab5a or Rab7a protein sequences were aligned to identify the amino acids to be mutated. Lamp1-RFP (Addgene) was cloned into pCS2+ vector. *Xenopus rps3a* and *rps4x* sequences were obtained by PCR from a *Xenopus laevis* cDNA library synthesized using SuperScript III First-Strand Synthesis System (ThermoFisher Scientific), and subsequently cloned into the N-terminal of GFP in pCS2+ vectors to construct Rps3a-GFP and Rps4x-GFP plasmids. *Xenopus* Vg1RBP was obtained from pET21d-Vg1RBP-GFP by PCR and cloned into a GFP-containing pCS2+ vector, which has been previously described (Leung et al., 2006). Mitochondria-targeted GFP (Mito-GFP) was a gift from Michael Coleman (Department of Clinical Neuroscience, University of Cambridge, UK). *Xenopus laminb2 (lmnb2)* cDNA sequence including the 5′ and 3′ untranslated regions (UTRs) was obtained from IMAGE clone (Source Bioscience SB5157193) by PCR and cloned into pCS2+ vector for Cy5-labeled mRNA synthesis. 24 SunTag tandem repeats were obtained from pcDNA4TO-24xGCN4_v4-kif18b-24xPP7 (Addgene) by PCR and cloned into pCS2+ vector. *lmnb2* 5′UTR was cloned into 5′ end of the SunTag repeats, while *lmnb2* coding sequence (CDS) and 3′UTR were cloned into 3′ end of the SunTag repeats, generating the *lmnb2* 5′UTR-24xSunTag-*lmnb2* CDS-*lmnb2* 3′UTR construct, which was used to generate Cy5-labeled SunTag-*lmnb2* mRNA. scFv-sfGFP sequence obtained from pHR-scFv-GCN4-sfGFP-GB1-dWPRE (Addgene) by PCR was cloned into pCS2+ vector. The E.coli DH5α strain was used for all plasmid amplification steps. Capped RNAs were *in vitro* transcribed using mMessage mMachine SP6 Transcription Kit (ThermoFisher Scientific). In case of Cy5-labeled mRNA synthesis, 1μl of Cy5-UTP (PerkinElmer) was added to the reaction mixture during *in vitro* transcription.

#### Blastomere Microinjection

DNA and RNA were microinjected into both of the dorsal blastomeres at four- or eight-cell stage as previously described (Leung and Holt, 2008). Embryos were de-jellied with 2% cysteine (Sigma-Aldrich) in 1X MBS (pH 8), washed 3 times in 0.1X MBS and aligned on a grid in 4% Ficoll (Sigma-Aldrich) in 0.1X MBS, 1% penicillin (100 U/ml), streptomycin (100 μg/ml) and fungizone 0.25 μg/ml (Antibiotic-Antimycotic, GIBCO). Injections of 5 nL of volume were performed using glass capillary needles (outer diameter: 1.0 mm; inner diameter: 0.5 mm, Harvard Apparatus) and a microinjector (Picospritzer, General Valve). Cy3-UTP (PerkinElmer) was injected at 100μM. RNA coding for GFP and GFP- or RFP-tagged wild-type and mutant Rab5a were injected at a concentration of 100ng/μl. RNA coding for GFP and GFP- or RFP-tagged wild-type and mutant Rab7a were injected at a concentration of 200ng/μl. Mito-GFP and GFP-Vg1RBP DNA plasmids were injected at a concentration of 25ng/μl. Lamp1-RFP DNA plasmid was injected at a concentration of 50ng/μl.

#### Targeted Eye Electroporation

DNA plasmids [1μg/μl] or *in vitro* transcribed RNAs [1μg/μl] were introduced into *Xenopus* eye primordia of stage 26-30 embryos by electroporation (Falk et al., 2007). Anaesthetized embryos were transferred to a “†” shaped chamber with the head positioned at the cross intersection and platinum electrodes positioned on either side, in the transverse channel. A borosilicate glass capillary needle (outer diameter: 1mm; inner diameter: 0.78mm, Harvard Apparatus) containing the DNA or RNA solution was inserted into the eye and a volume of 40nl was injected. The capillary was withdrawn from the eye post-injection, immediately prior to delivering eight square wave electric pulses of 18V, with 50ms duration and 1000ms intervals. After electroporation, embryos were transferred to 0.1x MBS for recovery and electroporated eyes were cultured at stage 35/36. For live-imaging experiments, Rps3a-GFP or Rps4x-GFP DNA plasmids were electroporated into RFP-Rab5a or RFP-Rab7a RNA- or Lamp1-RFP DNA-injected embryos. *In vitro* transcribed Cy5-labeled *lmnb2* mRNA was electroporated into GFP-Rab5a or GFP-Rab7 RNA-injected embryos.

#### Live Imaging in *Xenopus* Retinal Ganglion Cell Axons

Cultured axons were imaged under a Perkin Elmer Spinning Disk UltraVIEW ERS, Olympus IX81 inverted microscope with a 60x 1.4NA silicone oil objective for 2 to 5 minutes with exposure times set between 300-500ms. Volocity (Perkin Elmer) was used for manual tracking of Rab7a or Rab5aendosomes associated with either Cy3-RNA labeled granules or GFP-Vg1RBP. Associated signals were defined by their association/co-movement for > 4 s. A granule was defined as static or oscillatory if no directional transport further than 2μm within 60 s was observed. To visualize ER, retinal cultures were incubated with 1μM ER-Tracker Red (ThermoFisher Scientific) for 15 minutes and washed 7 times with culture medium. To visualize acidic endosomes, retinal cultures were incubated with 50nM Lyso-Tracker Red (ThermoFisher Scientific) for 30 minutes and washed 7 times with culture medium. Movies of Rab7a endosomes and mitochondria were taken for 5 minutes directly after the cultures were incubated for 20 minutes in 50nM MitoTracker red CMXRos (ThermoFisher Scientific) dissolved in DMSO and washed 7 times with culture medium. To quantify mitochondria membrane potential, retinal cultures were incubated with 20nM tetramethylrhodamine methyl ester (TMRM) (ThermoFisher Scientific) for 20 minutes, followed by 4 washes and immediate image acquisition of single frames with constant laser power and exposure time. Object detection analysis on Volocity was then used to quantify the mean intensity of the TMRM signal in each mitochondrion present in 50μm of isolated axon shafts expressing the indicated constructs, excluding the last 20μm distal portion of the axon. The mitochondria outlines were then placed in an adjacent area clear of mitochondria to record the background fluorescent intensity. This reading was subtracted yielding the background-corrected intensity.

#### Cy3-RNA Granules and LysoTracker Vesicles Analysis

Particle movements were extracted from raw data movies with the plusTipTracker package (Applegate et al., 2011). We used the Watershed-based option in the Detection Settings for all movies. To detect individual features (granules), movies were denoised with 2 Gaussian Kernels of size 1 pixel and 3 pixels respectively, corresponding to the sigma 1 & 2 in the Detection Settings of the plusTipTracker package. The K-Value (local threshold) was set to values between 2-5 depending on the background noise in the movies. The Tracking settings were held constant for all movies: Search Radius Range = 3-10, Minimum Sub-Track length = 4 frames (to discard background fluctuations), Maximum Gap Length = 3 (frames for which a particle is not detected due to overlap with another particle), Max Shrinkage Factor = 0, Maximum angle Forward/Backward = 30/0, Fluctuation Radius = 1.5. After running this analysis, several tracks (x-y coordinates) were generated for each movie.

The tracks representing Cy3-RNA granule or LysoTracker vesicle movements in each movie were then post processed in a self-written MATLAB script to determine their motion types. First, a segmented line was drawn along the axon toward the anterograde direction. Then each (average) track direction was calculated. If the average direction points within 162 degrees of the forward/anterograde direction of the drawn axon, the track is classified as anterograde and vice versa for retrograde. Furthermore, tracks that displace less than 2μm from their origin were classified as oscillatory. To calculate the Cy3-RNA track/granule intensity a background region was drawn by hand and the average pixel intensity in this region subtracted from the average intensity of the granule for each track (Individual granule intensities are calculated in the detection part of the plusTipTracker script). The average track velocity was calculated as the average of all frame-to-frame velocities.

#### Pixel Intensity, Area of Coverage and Particle Distance Analysis

Images of fluorescently labeled RNA granules, Rab5a endosomes, Rab7a endosomes, or mitochondria were analyzed using custom-written scripts within the MATLAB environment. All grayscale fluorescent images were binarized using Otsu’s thresholding method (Otsu, 1979). The binary images were segmented using a modified watershed method to obtain masks of labeled granules from each fluorescent channel. The resulting images were then visually inspected and the threshold was adjusted where granules were misidentified.

The masks from the Cy3-RNA channel were compared spatially to each of the other three and the granules that overlapped with GFP-Rab5a, GFP-Rab7a or mitochondria, by at least one pixel, were identified. The Cy3-RNA granules were thus grouped as in contact or not in contact with the other component. The area of each granule was defined as the total number of pixels of its mask and was normalized to the smallest and largest granule areas within one image (typically of a single axon). Since the mask areas of brighter granules tended to be larger, we calculated the mean intensities of the brightest pixels within each mask to remove any possible dampening of the signal due to mask size. The number of brightest pixels to be included was separately determined for each of Cy3-RNA granule pairs and set as half of the average area of Cy3-RNA granule masks in that pair. Pixel intensities of the granules were obtained from the original grayscale image and normalized such that the minimum value would equal the binarization threshold and the maximum value was that of the brightest pixel within the corresponding image’s masks.

The masks from RFP-Rab7a were compared to those from mitochondria to find the distances between objects in each image. Where an overlap between two objects from each channel existed, the distance between those objects was set to zero. Otherwise, the shortest distance between an object and its nearest neighbor in the other channel was calculated. These shortest distances for all objects were collated and analyzed. To create a randomized distribution of endosomes and mitochondria for comparison, the mean and standard deviations of their mask radii, number of particles, and axon dimensions were calculated. Normal distributions based on these mean and standard deviations were created for each of the above variables. Random numbers of each particle type were drawn from normal distribution of particle numbers. Each particle was a circle of random radius drawn from the respective normal distribution. Particles were placed in axons whose width and length were drawn from corresponding distributions, as well. The distance between these randomly selected particles were measured and analyzed as described above.

#### Immunoprecipitation

For GFP immunoprecipitations, GFP, GFP-Rab5a or GFP-Rab7a expressing *Xenopus* brains were dissected from stage 35/36 embryos (70 embryos/condition) and homogenized in lysis buffer (20mM Tris, 100mM NaCl, 10mM MgCl2, 0.25% NP40, 10% Glycerol, 100 μg/ml CHX (Sigma-Aldrich)) supplemented with EDTA-free protease inhibitor cocktail (Roche), phosphatase inhibitor (Pierce) and 100U/ml SUPERase In RNase inhibitor (Ambion) for 5 minutes on ice. Following centrifugation for 5 minutes at 1000 x g, the supernatant was collected. For immunoprecipitation, the protein extracts were incubated for 30 minutes at 4°C with beads coupled to an anti-GFP antibody (ab1218, Abcam). Beads were washed four times with lysis buffer and samples were either eluted from the beads by adding RLT buffer (QIAGEN) and vortexing for 2 minutes for subsequent RNA isolation or eluted with 1x SDS sample buffer for 5 minutes at 95°C for protein and subsequent western blot analysis as described below. For endogenous immunoprecipitations, brains from stage 35/36 embryos (70 embryos/condition) were dissected and lysed in lysis buffer as described above. Lysates were then incubated with beads coupled to anti-Rab7 (ab77993, Abcam) or IgG control (ab37415, Abcam) antibodies for 30 minutes at 4°C. Beads were washed three times with lysis buffer and samples were eluted from the beads with 1x SDS sample buffer for 5 minutes at 95°C for protein and subsequent western blot analysis.

#### Quantitative PCR

RNA was isolated from eluted samples using the RNeasy Mini kit (QIAGEN) and reverse transcribed into cDNA using random hexamers and the SuperScript III First-Strand Synthesis System (ThermoFisher Scientific). The cDNA was used to prepare triplicate reactions for qPCR according to manufacturer’s instructions (QuantiTect SYBR Green PCR kit, QIAGEN) and plates were centrifuged shortly and run on a LightCycler 480 (Roche) using the following PCR conditions: denaturation for 15 s at 94°C; annealing for 30 s at 60°C; extension for 30 s at 72°C. The levels for each condition were corrected with their own input. The following primers were used for qPCR: for *β-actin*, 5′-CCAGAAGAACACCCAGTGCT-3′ and 5′-CAGGGACAACACAGCTTGGA-3′; for *lmnb2*, 5′-GCAAGTGAAGATGTACAAGGAAGAA-3′ and 5′-CGTCGCTCAGTTAATTCTTCTAGGG-3′; for *vdac2*, 5′-ACTCGCATGCAGCCATATCT-3′ and 5′-TACCAGCGCAGAAAGTGTGA-3′.

#### Western Blot

Proteins were resolved by SDS-PAGE on NuPage 4%–12% Bis-Tris gels (ThermoFisher Scientific) and transferred to nitrocellulose membrane (Bio-Rad). The blots were blocked in milk for 45 minutes at room temperature and then incubated with primary antibodies in milk overnight at 4°C. After washing 3 times with TBS + 0.1% Tween (TBS-T) the blots were incubated with HRP-conjugated secondary antibodies for 1 hour at RT, washed again for 3 times in TBS-T, followed by ECL-based detection (Pierce ECL plus, ThermoFisher Scientific). The following primary antibodies were used for western blot analysis: rabbit anti-Rab7 (ab137029, Abcam), rabbit anti-Rpl10A (16681-1-AP, Proteintech), mouse anti-Rps3A (ab194670, Abcam), mouse anti-FXR (gift from Dr. Khandjian), rabbit anti-SFPQ (ab38148, Abcam), rabbit anti-Vg1RBP (gift from Dr. Standart), rabbit anti-mTOR (#2983, Cell Signaling).

#### Immunohistochemistry

Retinal cultures were fixed in 2% formaldehyde/7.5% sucrose in PBS for 20 minutes at 20°C. Antigen retrieval by steaming the fixed cultures in 0.01M sodium citrate (0.05% Tween, pH 6.0) for 10 minutes was carried out before staining for phospho-AKT (Ser473), phospho-mTOR (Ser2448) and phospho-S6 (Ser235/236). The cultures were then washed 3 times in PBS + 0.001% Triton-X-100, permeabilized for 3-5 minutes in 0.1% Triton-X-100 in PBS, or 25 minutes in 0.1% Saponin (for LAMP-1 staining), washed 3 times in PBS + 0.001% Triton-X-100 and blocked with 5% heat-inactivated goat serum in PBS for 30 minutes at 20°C. Primary antibodies were incubated overnight, followed by Alexa Fluor-conjugated secondary antibodies for 45 minutes at 20°C in dark. Cultures were mounted in FluorSave (Calbiochem) or Vectashield (Vector Laboratory) for super-resolution microscopy. Antibodies were used at the following dilutions. 1:200 rabbit anti-RPS3A (14123-1-AP, Proteintech), 1:200 mouse anti-RPS3A (ab194670, Abcam), 1:200 mouse anti-RPL19 (ab58328, Abcam), 1:200 rabbit anti-RPL24 (17082-1-AP, Proteintech), 1:400 rabbit anti-Vg1RBP (gift from Dr. Standart), 1:250 rabbit anti-LAMP-1 (ab24170, Abcam), 1:400 mouse anti-FXR (gift from Dr. Khandjian), 1:50 mouse anti-Rab5 (sc-46692, Santa Cruz), 1:300 rabbit anti-Rab7 (ab137029, Abcam), 1:50 mouse anti-GM130 (BD610822, BD Biosciences), rabbit anti-Rpl10A (16681-1-AP, Proteintech), 1:100 rabbit anti-phospho-AKT (Ser473) (9271T, Cell Signaling), 1:200 rabbit anti-phospho-mTOR (Ser2448) (ab109268, Abcam), 1:200 rabbit anti-mTOR (#2983, Cell Signaling), 1:800 rabbit anti-phospho-S6 Ribosomal Protein (Ser235/236) (#2211, Cell Signaling), 1:200 rabbit anti-SFPQ (ab38148, Abcam). Secondary antibodies: 1:500 goat anti-mouse/rabbit Alexa Fluor 488 (ThermoFisher Scientific) or 1:500 goat anti-mouse rabbit Alexa Fluor 568 (ThermoFisher Scientific), except 1:1600 goat anti-rabbit Alexa Fluor 568 for phospho-S6, 1:1000 goat anti-rabbit Alexa Fluor 568 (ThermoFisher Scientific) for SFPQ immunohistochemistry.

#### Molecular Beacon Live Imaging

To visualize endogenous *β-actin* mRNA in *Xenopus laevis* RGC axons, two molecular beacons (MBs) were designed to target predicted single stranded regions in *β-actin* mRNA (Turner-Bridger et al., 2018). Briefly, the MBs were made of nuclease-resistant nucleotides which were either LNA (+N) or 2′-*O*-methyl RNA (oN) bases, ending with Cy3:BHQ-2™ fluorophore:quencher pair combination. Probe sequences were: MB1 Cy3-oCoGoAoCoGoCoU+CoAoGoUoU+AoGoG+AoUoUoUoUoC+AoUoGoCoGoUoCoG-BHQ2; MB2 Cy3-oGoCoGoCoAoG+GoAoA+GoCoCoAoA+GoAoUoG+GoAoUoGoCoGoC-BHQ2. MB1 and MB2 were electroporated at a concentration of 25 μM into stage 28 eye primordia together with either GFP-Rab5a or GFP-Rab7a *in vitro* transcribed mRNA (1 μg μl^-1^). Eyes were dissected and cultured when *Xenopus* embryos reached stage 32, and RGC axons imaged the next day. Images were acquired on an Olympus IX81 inverted microscope that was fitted with a PerkinElmer Spinning Disk UltraVIEW VoX using a 100x oil immersion objective (1.4 N.A., Nikon), and an ORCA-Flash4.0 V2 CMOS camera (Hamamatsu). Volocity 6.3.0 software (PerkinElmer) was used for acquisition. Images were acquired at a frame rate of 1.5 s for 30 s. *β-actin* mRNA and either GFP-Rab5a or GFP-Rab7a were classed as associated if the signal from the GFP (488nm laser line) and MB-Cy3 (561nm laser line) channels merged, and remained merged for the duration of the movie, or until one of the puncta bleached (minimum 5 frames). To avoid bias in the degree of observed association due to variable levels of either MBs or GFP-Rabs proteins in axons, which was introduced by electroporation, we analyzed colocalization within 25 μm sections of axons that contained 0.8:1.5 ratio of MB:GFP-Rabs puncta. Associated puncta were defined as “oscillatory,” if they remained within a 2 μm radius for the duration of the movie, or as “moving,” if the total distance traveled was greater than 2 μm.

#### Axon-only Puromycin Labeling Assay

After 48 hours, axons were severed from their cell bodies and subsequently treated with 2 μM puromycin (Sigma-Aldrich) for 10 minutes. After treatment, the cultures were fixed in 2% formaldehyde/7.5% sucrose in PBS for 20 minutes at RT, washed 3 times in PBS + 0.001% Triton-X-100, permeabilized for 3-5 minutes with 0.1% Triton-X-100 and blocked in 5% heat-inactivated goat serum in PBS for 30 minutes at RT and subsequently labeled with Alexa Fluor 647-conjugated mouse anti-puromycin antibody (1:250, MABE343-AF647, Millipore) overnight. For experiments involving pharmacological treatments, axons were pre-treated with 50μM CHX (Sigma-Aldrich) for 20 minutes, 50μM dynasore (Sigma-Aldrich) for 20 minutes, 50μM Chloroquine (Sigma-Aldrich) or 2.5μM PP242 for 10 minutes before puromycin administration.

#### Quantification of Immunofluorescence

Randomly selected non-collapsed growth cones were imaged at 60x on a Nikon Eclipse TE2000-U inverted microscope equipped with an EMCCD camera. Exposure time was kept constant and below gray-scale pixel saturation. For quantitation of fluorescence intensity, the growth cone outline was traced on the phase contrast image using Volocity (PerkinElmer), then superimposed on the fluorescent image. The software calculated the fluorescent intensity within the growth cone, giving a measurement of pixel intensity per unit area. The growth cone outline was then placed in an adjacent area clear of cellular material to record the background fluorescent intensity. This reading was subtracted from the growth cone reading, yielding the background-corrected intensity. To plot the signal intensities, pixel fluorescent intensity across a line segment was measured in ImageJ. For colocalization test, Pearson’s correlation coefficient was obtained in ImageJ by using coloc2 plugin. Kymograph were obtained in ImageJ by using Multi kymograph plugin.

#### SunTag-lmnb2 Imaging

For SunTag-*lmnb2* imaging, *in vitro* synthesized scFv-GFP and RFP-Rab7a mRNAs (200ng/μl) were injected (5nl) into each of the two dorsal blastomeres at 4-cell stage. When the embryos developed to stage 26, Cy5-labeled SunTag-*lmnb2* mRNA (1μg/μl) was electroporated into the retinal primordia. When the embryos reached stage 35/36, electroporated eyes were dissected and cultured. After 24 hours, continuous imaging of distal axons expressing *in vitro* synthesized scFv-GFP and RFP-Rab7a mRNAs was performed under a Perkin Elmer Spinning Disk UltraVIEW ERS, Olympus IX81 inverted microscope with a 100x oil objective and an ORCA-Flash4.0 camera (Hamamatsu) for 1 minute. Exposure times were set between 50-500ms for each channel. Laser power of 488, 559 and 640nm laser line was set to 10%–30% of maximal intensity. Time-lapse images were recorded by Volocity software. In pharmacologically treated conditions, the retinal cultures were incubated with 100 μg/ml puromycin or 50μM chloroquine for 1h before imaging.

#### Proximity Ligation Assay

This experiment was carried out according to the manufacturer’s protocol (Duolink Biosciences) with specific modifications (Yoon et al., 2012). Eyes were severed from axonal explants after 48 h of outgrowth. Cultures were treated with 2 μM puromycin for 10 minutes at RT, fixed in 2% formaldehyde/7.5% sucrose in PBS for 20 minutes at 20°C, washed 3 times in PBS + 0.001% Triton X-100, permeabilized for 3-5 minutes in 0.1% Triton X-100 in PBS, washed three times in PBS, blocked with 5% heat-inactivated goat or horse serum in PBS for 30 minutes at RT and subsequently incubated with primary antibodies overnight at 4°C. Primary antibodies were diluted at 1:500 for unconjugated mouse anti-puromycin (MABE343, Millipore), 1:200 for rabbit anti-Lamin B2 (ab97513, Abcam) and 1:500 for goat anti-VDAC2 (ab37985, Abcam). Dishes were washed twice for 5 minutes with 0.002% Triton X-100 in PBS and incubated with anti-rabbit (+) or anti-goat (+) and anti-mouse (-) PLA probes for 1 hour at 37°C, with ligase for 30 minutes at 37°C and with the polymerase mix with red fluorescence for 100 min at 37°C. The samples were subsequently mounted with the mounting medium (DUO82040, Duolink) and imaged using a Nikon Eclipse TE2000-U inverted microscope equipped with an EMCCD camera. The number of discrete fluorescent puncta within a randomly chosen 20μm axon segment in each image was counted using Volocity.

#### OMX Super-resolution Microscopy

Super-resolution 3D SIM images were acquired using a Deltavision OMX 3D SIM System V3 from Applied Precision (a GE Healthcare company) equipped with 3 EMCCD Cascade cameras from Photometrics, 488nm and 592.5 nm diode laser illumination, an Olympus PlanSApo 100 × 1.40 NA oil objective, and standard excitation and emission filter sets. Imaging of each channel was performed sequentially using three angles and five phase shifts of the illumination pattern as described (Gustafsson et al., 2008). The refractive index of the immersion oil (Cargille) was adjusted to 1.513 to minimize spherical aberrations. Sections were acquired at 0.1 μm z steps. Raw OMX data were reconstructed and channel registered in the SoftWoRx software (Applied Precision, a GE Healthcare company). Reconstructions were carried out using channel-specific Optical Transfer Functions (OTFs) and channel-specific K0 angles. OTFs were generated within the SoftWoRx software by imaging 100 nm beads (Life Technologies) using appropriate immersion oils to match the data. Channel registration was carried out using the Image Registration parameters generated within the SoftWoRx software and checked for accuracy by imaging Tetraspeck beads (Life Technologies). Channel registration was accurate to one pixel. Further data analysis was performed using Fiji.

#### Retinal Projection Visualization and Analysis

Anaesthetized embryos of either sex were fixed at stage 39 or 45 in 4% formaldehyde in PBS at 4°C overnight and washed 3 times with filtered PBS. RGC axons of one eye per embryo were labeled by intraocular injection of fluorescent lipophilic dye, DiI (ThermoFisher Scientific). Embryos were left in PBS for 48 hours allowing the diffusion of the dye into the optic tract. The contralateral (or non-dye injected side) brain hemisphere was later dissected, mounted in PBS and visualized using confocal under 559nm laser line. Brains with less than 30 visible axons were categorized as defective axonal projection phenotypes. For single-blastomere injection experiments, axonal projections were analyzed by using Volocity automated detection of the fluorescence signal, creating a 3D mask matching the signal and providing the corresponding volume.

### Quantification and Statistical Analysis

All experiments were performed in at least three independent biological replicates. The n number for each experiment, details of statistical analysis and software are described in the figure legends or main text. Statistical analyses used in this study include Wilcoxon Ranksum Test, Mann-Whitney test and Fisher’s exact Test. Statistical significance is defined as, n.s., not significant, ^∗^p < 0.05, ^∗∗^p < 0.01, ^∗∗∗^p < 0.001. Statistical analysis was performed using Prism (GraphPad) or MATLAB (MathWorks).

## Appendix

Supplemental Information includes five figures and five videos and can be found with this article online at https://doi.org/10.1016/j.cell.2018.11.030.
